# Nucleic acid aptamers: new methods for selection, target validation, molecular diagnostics and therapeutics

**DOI:** 10.1038/s41392-026-02930-y

**Published:** 2026-09-07

**Authors:** Ding-Kun Ji, Haozhi Lei, Jia Liu, Fangfang Xia, Xiaobo Gao, Yi-Qing Zhou, Ruiying Li, Yongqi Han, Ruowen Wang, Weihong Tan

**Affiliations:** 1 Institute of Molecular Medicine (IMM), Shanghai Key Laboratory for Nucleic Acid Chemistry and Nanomedicine, Renji Hospital, School of Medicine, Shanghai Jiao Tong University, Shanghai, China; 2 Medical and Engineering Cross Research Institute, The First Affiliated Hospital of Henan University, Henan University, Kaifeng, China; 3 Department of Anatomy and Physiology, Shanghai Jiao Tong University School of Medicine, Shanghai, China; 4 Zhejiang Cancer Hospital, Hangzhou Institute of Medicine (HIM), The Chinese Academy of Sciences, Hangzhou, Zhejiang China

**Keywords:** Molecular medicine, Drug development

## Abstract

Nucleic acid aptamers, often referred to as “chemical antibodies,” are versatile, specific, and easily modifiable functional nucleic acids. There is a growing focus on new methods for the selection and target validation of aptamers, with the aim of expanding their biomedical applications in molecular diagnostics and therapeutics, which is currently a research hotspot. This review is composed of eight sections. In the first section, we briefly introduce aptamers and review their development in molecular diagnostics and therapeutics. The “Advantages of aptamers in molecular diagnosis and therapeutics” section summarizes and discusses the advantages of aptamers in these fields. The “New methods for screening aptamers” section presents and discusses nucleic acid aptamer screening methods, including both classical and novel approaches. In the “New methods for target validation” section, we explore new methods for target validation, covering aptamer structure validation, target recognition validation, and aptamer–target interaction validation. The “New methods for molecular diagnostics” section summarizes and discusses recent applications of aptamers in molecular diagnostics, particularly focusing on new mechanisms and detection strategies as well as their applications in various diseases. The “New methods for molecular therapeutics” section summarizes and discusses recent applications of aptamers in molecular therapeutics, emphasizing new mechanisms and aptamer-based therapy strategies, along with their therapeutic applications in different diseases. The “Challenges and future perspectives of nucleic acid aptamers” section addresses the challenges and future perspectives of aptamers in disease diagnosis and treatment. Finally, the “Conclusion” section shares our views on the future directions of aptamers in clinical disease molecular diagnostics and therapeutics.

## Introduction

Nucleic acid aptamers are short, single-stranded deoxyribonucleic acid (ssDNA) or ribonucleic acid (RNA) molecules that can bind to target molecules.^1,2^ In 1990, they were independently reported by the laboratories of Joyce^3^ and Szostak^4^ as well as Gold.^5^ Aptamers are selected through the process known as ‘systematic evolution of ligands by exponential enrichment’ (SELEX), which is an iterative selection method that utilizes large libraries of nucleic acids. During this process, aptamer sequences that bind to target molecules are enriched. Candidate binding sequences undergo multiple selection rounds to increase the population of high-affinity species until they dominate the library.^6–10^ The term ‘aptamer’ was coined by Ellington, derived from the Latin word aptus (meaning ‘to fit’) and the Greek word meros (meaning ‘part’).^4^ Aptamers are one of the few classes of molecules, alongside antibodies, that can be engineered to bind to multiple different targets. The introduction of SELEX against live targets, including living cells (cell-SELEX),^6,11–13^ tissue samples (tissue-SELEX),^14–16^ and whole organisms (whole organism in vivo SELEX),^17,18^ marked a significant advancement in aptamer discovery. To date, thousands of aptamers have been generated against a wide range of targets, including proteins, peptides, organic molecules, small metal ions, viruses, bacteria, whole cells, and even targets within live animals.^19,20^

Nucleic acid aptamers possess unique tertiary structures, enabling them to specifically bind to their cognate molecular targets.^21,22^ They are often referred to as “chemical antibodies” due to their ability to target and bind in a manner similar to traditional antibodies. However, aptamers are more economical and easier to synthesize while retaining properties comparable to those of antibodies.^23,24^ Additionally, the aptamer selection process is generally faster than that for monoclonal antibodies and allows for the optimization of binding affinity through successive rounds of evolutionary screening and modification. The ability of aptamers to be easily assembled into multifunctional complexes with active drugs or probes makes them particularly attractive as “magic bullets” for precision medicine.^13,25,26^ Various aptamer-based molecular diagnostic platforms have been widely developed for disease diagnosis,^27–30^ infectious disease prevention and control,^31–35^ point-of-care testing (POCT)^36–38^ and wearable diagnostic devices.^39–43^ Furthermore, aptamers can specifically deliver small chemotherapy drugs, oligonucleotides, peptides, proteins, and even “live drugs” to targeted cells and tissues for efficient molecular therapeutics.^44–47^ Many clinical diseases, including cancer,^23,26,48^ cardiovascular diseases,^49,50^ and neurodegenerative diseases^51,52^, have benefited from aptamer-based molecular therapies.

Although aptamers have been studied for nearly 40 years, dating back to the early 1990s, interest from academia and private investors has lagged behind that in other nucleic acid-based therapeutics and monoclonal antibodies (mAbs) until recently.^20,23^ Currently, only two aptamer drugs are approved by the U.S. Food and Drug Administration (FDA). The first is pegaptanib sodium (Macugen), an RNA aptamer drug that targets vascular endothelial growth factor (VEGF) with high binding affinity. It was approved by the FDA for the treatment of age-related macular degeneration, not for cancer, in 2004.^53^ The second RNA aptamer drug, avacincaptad pegol (Izervay), was approved in 2023 for geographic atrophy (GA) secondary to age-related macular degeneration.^54^ Two major obstacles common to all nucleic acid-based therapeutics initially hindered the use of aptamers as therapeutic drugs: the immunogenicity of natural oligonucleotides and their high sensitivity to serum nucleases.^55–58^ To address these issues, intensive follow-up studies led to the development of aptamers with specific chemical modifications in the nucleotides and/or backbone, which significantly reduced immunogenic potential and increased resistance to enzymatic degradation from a few minutes to several days.^24,48,59^ These modifications have enhanced the clinical applicability of aptamers. Since then, additional factors, such as the explosive development of coronavirus disease 2019 (COVID-19) vaccines and tumor vaccines, have spurred interest in aptamer therapeutics.^23^ According to clinical trial data (ClinicalTrials.gov), as of March 15, 2026, more than 71 aptamer-related reagents have entered clinical testing. These efforts have accelerated the development and clinical implementation of aptamer-based molecular diagnostics and therapies.

**Fig. 1:**
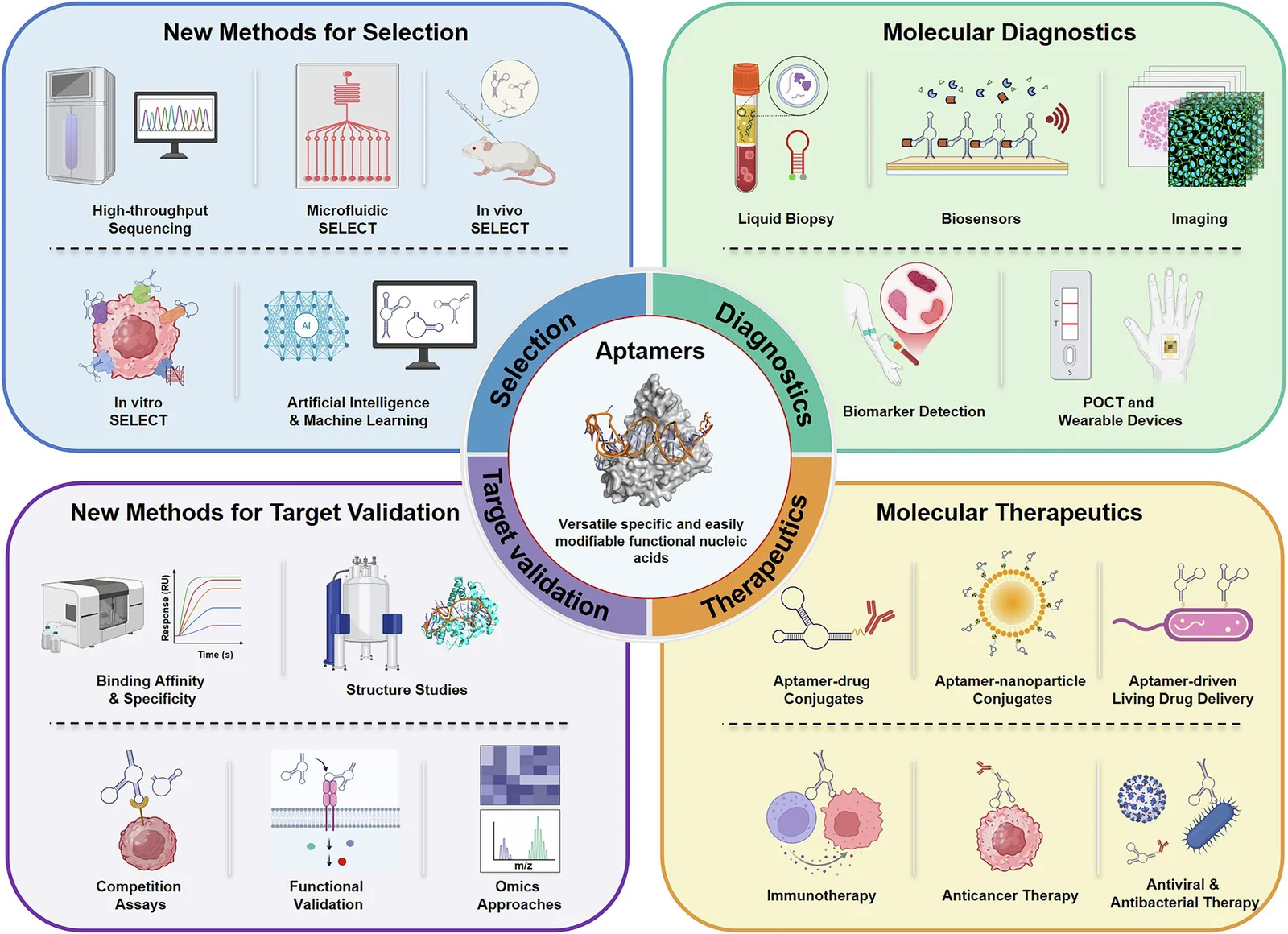
Summary of new methods for the selection, target validation, molecular diagnostics, and therapeutics of nucleic acid aptamers. The new methods for selection include high-throughput sequencing, microfluidic SELECT, in vivo SELECT, in vitro SELECT, and artificial intelligence & machine learning. The new methods for target validation include binding affinity & specificity tests, structure studies, competition assays, functional validation, and omics approaches. The molecular diagnostics of aptamers include liquid biopsy, biosensors, imaging, biomarker detection, and POCT & wearable devices. The molecular therapeutics of aptamers include aptamer-drug conjugates, aptamer-nanoparticle conjugates, aptamer-driven living drug delivery, immunotherapy, anticancer therapy, and antiviral & antibacterial therapy. Some resources in this figure come from BioRender

This review focuses on the technological advances of the past five years in the selection and target validation of aptamers, as well as their applications in molecular diagnostics and therapeutics (Fig. 1). It will also address the challenges and future perspectives of aptamers in disease molecular diagnosis and treatment.

## Advantages of aptamers in molecular diagnosis and therapeutics

The human body is a complex system composed of countless molecular machines working in a highly ordered and coordinated manner.^60,61^ Abnormal molecular states are the primary cause of disease occurrence and development. Abnormal molecules, such as mutated genes, overexpressed membrane proteins, and abnormally secreted proteins, have become key targets for disease diagnosis and treatment.^62^ Molecular diagnostics and therapeutics are integral to cutting-edge precision medicine. Also known as personalized medicine, precision medicine is an emerging approach to healthcare that tailors medical treatments to the individual characteristics of each patient.^63,64^ Rather than relying on a one-size-fits-all approach, precision medicine considers factors such as a patient’s genetics, environment, and lifestyle to deliver more targeted and effective care.^65,66^ Nucleic acid aptamer-based molecular tools play a crucial role in precision medicine and are attracting increasing attention.

As one of the most developed molecular tools, antibodies have been explored for over a century and are suited for various molecular diagnostic and therapeutic applications.^67,68^ Antibodies and antibody‒drug conjugates (ADCs) have become the most popular strategies for disease detection and treatment. To date, more than 80 antibodies and 20 ADCs have been approved and are available on the market.^69–71^ While antibody technology plays a vital role in diagnostics and therapeutics, it is not without limitations. Some of these limitations are becoming increasingly apparent and may restrict their use in various fields. For example, (1) Antibodies must be biologically produced, which is inevitably costly. (2) The selection of antibodies for lipids, carbohydrates, and organic macromolecules has often resulted in mediocre affinity and specificity, highlighting the need for novel targeting ligands.^72^ (3) Their large size usually slows tissue penetration. (4) A critical criterion for covalently conjugating antibodies with drugs is the preservation of their affinity and specificity for their receptors.^73^ (5) There are nonspecific conjugation sites for functional molecules on antibodies. (6) Other intrinsic issues exist, such as limited conjugation numbers of payloads, leading to high costs as well as tremendous time and effort.^70,74^

Aptamers are a class of artificial ligands that expand the repertoire of potential targets, including small molecules and ions, during the selection protocol (Table 1).^1,19,75^ Aptamers have a small molecular weight (5–15 kDa) compared to antibodies (150–1000 kDa) and nanobodies (12–15 kDa), resulting in a higher binding density.^76^ Their smaller size allows them to move more rapidly than larger antibodies, encountering biomembrane structures more frequently and diffusing more easily into binding sites due to fewer steric restrictions and relatively higher diffusion rates.^77^ (2) Biosafety advantage: Being small and nucleic acid-based, aptamers exhibit little to no toxicity or immunogenicity. Clinical and preclinical trials have demonstrated that the human body can tolerate micromolar levels of oligonucleotides, and aptamers can penetrate deeper into tumor cores than antibodies.^10^ (3) Chemistry advantage: Aptamers are easier to chemically synthesize and modify than antibodies and nanobodies. They are suitable for large-scale synthesis using solid-phase technology, resulting in consistent quality with minimal batch-to-batch differences.^78^ (4) Target advantage: Aptamers have a broader target library than antibodies. They can be selected under native conditions for various targets, including metal ions, small organic molecules, proteins, sugars, lipids, and even whole cells or viruses.^23^ (5) Cost advantage: Aptamers can be chemically synthesized in vitro, making them quicker to produce and more economical. They are stable under harsh conditions, including high temperatures, and have a longer shelf life. Additionally, aptamers can be easily transported without the need for cold chain requirements, reducing costs associated with long-term storage and transport.^24,78^

**Table 1:** Comparative advantages and disadvantages of antibodies and aptamers

Feature	Antibodies	Aptamers
**Structure**	Proteins.	Nucleic acids.
**Production**	~6 months. Time-consuming, costly, batch variability.	~2 months. Fast, cheap, batch-to-batch stability.
**Size**	~150 kDa.	~5–15 kDa.
**Tissue Penetration**	Limited by large molecular size.	Excellent due to its small size.
**Stability**	Thermally sensitive (room temperature −37 °C); irreversible denaturation. Excellent in vivo pharmacokinetic stability (7~21 days).	High chemical stability; Thermally stable up to 95 °C with reversible folding. Poor in vivo pharmacokinetic stability. Rapid renal clearance (less than 30 min).
**Modifiability**	Site-specific conjugation is challenging and may disrupt folding and function.	Allows precise, stable modification at predefined sites during synthesis.
**Immunogenicity**	Risk of eliciting immune responses (HAMA).	Low immunogenicity.
**Affinity**	High (pM–nM range). (can reach subpM/fM after affinity maturation).	Comparable high affinity (0.1–300 nM) (occasionally reaching pM affinity).
**Target range**	Primarily proteins and peptides.	Extremely broad: proteins, small molecules, ions, cells, even entire organisms.

## New methods for screening aptamers

**Fig. 2:**
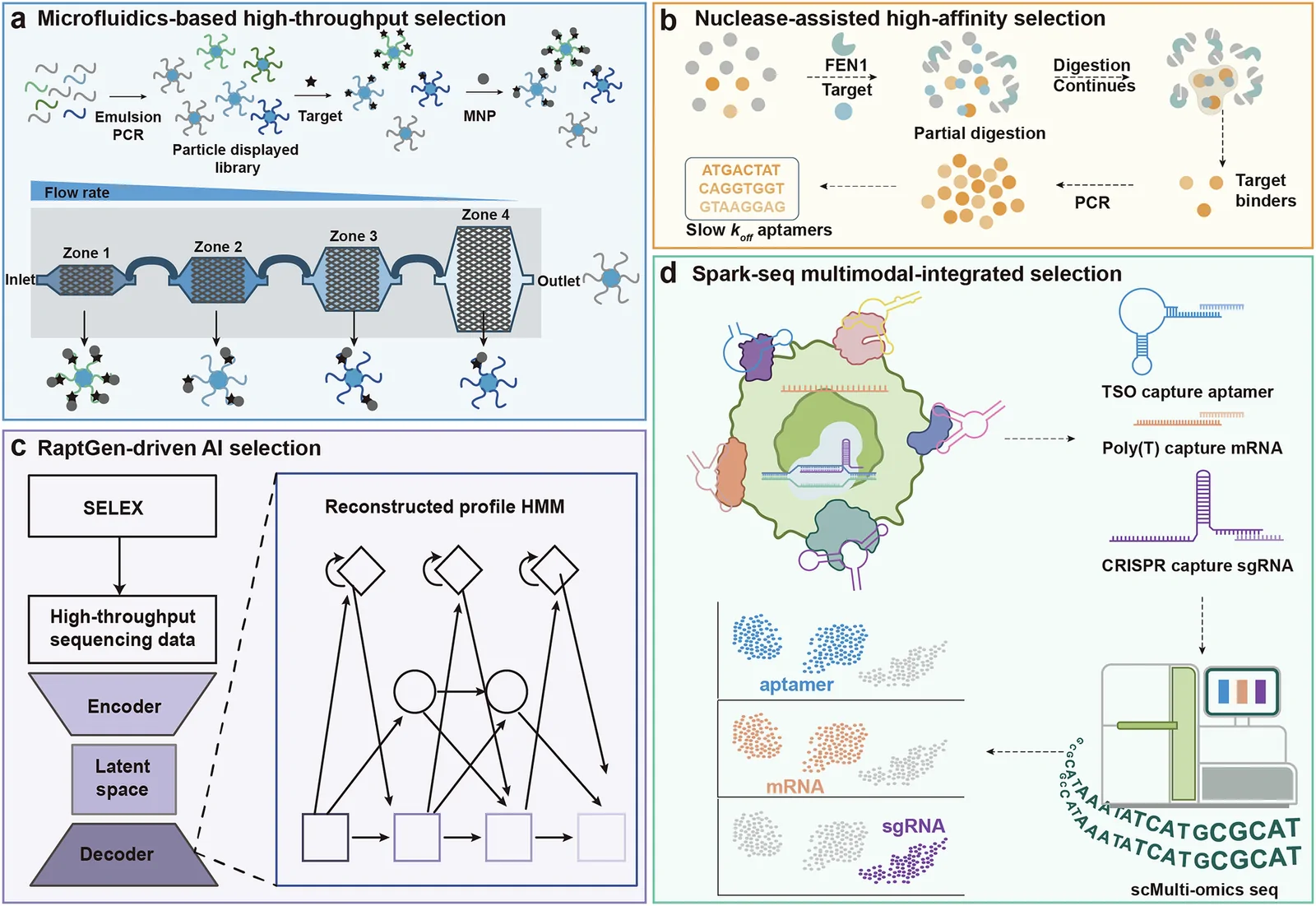
New methods for screening aptamers. **a** Pro-SELEX^81^ enhances high-throughput selection by incorporating monoclonal particle display with high-dimensional microfluidic sorting, enabling single-round discovery of aptamers and allowing for the precise mapping of binding performance across a broad kinetic range. **b** Nuclease-assisted strategies improve the selection of high-affinity aptamers by converting target-induced conformational switches into enzymatic resistance, where endo-^82^ or exonucleases^110,111^ act as stringent molecular sieves to selectively hydrolyze background sequences while retaining candidates with superior structural stability and slow dissociation rates. **c** RaptGen,^83^ as a transformative generative AI technology, streamlines aptamer discovery by integrating a Variational Autoencoder (VAE) with a profile Hidden Markov Model (HMM) decoder, enabling the proactive in silico generation and activity-guided optimization of novel candidates within a continuous latent space. **d** The SPARK-seq platform^84^ represents a pinnacle of multimodal integration by merging clustered regularly interspaced short palindromic repeats (CRISPR)-based genetic perturbation with single-cell multiomics, providing a high-resolution map of in situ binding kinetics and functional phenotypes, effectively transforming aptamer discovery into a data-driven pipeline for target identification

The exceptional recognition capabilities and versatile therapeutic potential of nucleic acid aptamers have catalyzed a burgeoning demand for robust discovery methodologies. However, the practical translation of aptamers has long been bottlenecked by the inherent limitations of traditional SELEX,^4,76,79^ including labor-intensive iterative cycles, insufficient binding affinity, and lack of biological functions.^6,75,80^ To overcome these challenges, a new generation of innovative screening platforms has recently emerged, marking a paradigm shift from empirical screening toward precision engineering (Fig. 2). The new representative screening technologies include Pro-SELEX,^81^ Nuclease-assisted strategies,^82^ RaptGen-based artificial intelligence (AI) technology,^83^ integrated single-cell perturbation-driven aptamer recognition and kinetics sequencing (SPARK-seq) platform.^84^ These methodologies evolve traditional protocols into superior throughput, enhanced affinity, and function-oriented screening. Furthermore, the incorporation of intelligent algorithms and multimodal integration has streamlined the path from initial library partitioning to final lead identification. Table 2 summarizes the advantages and limitations of recently advanced aptamer selection platforms.

**Table 2:** Summary of the advantages and limitations of recently advanced aptamer selection platforms

SELEX platforms	Representative technologies	Key advantages	Main limitations
High-throughput aptamer selection platform	1. Pro-SELEX: emulsion PCR+microfluidic sorting 2. UltraSelex: successive wash fractions 3. HAS: 3D nonfouling macroporous hydrogel	1. Shortens the multiple screening cycles (>8 cycles) to a single-round selection. 2. Realizes the rapid generation of aptamers with defined affinity. 3. Evolves into an instrument-free system with HAS.	1. Lacks of systematic optimization and in-depth mechanistic research. 2. High-throughput screening against living cells remains challenging.
High-affinity aptamer selection platform	1. Library design: active-site-guided epitope targeting, fixed motif incorporation, electrophilic warhead modification 2. Screening optimization: nuclease-assisted kinetic filtering 3. Functional design: Blocker-SELEX, Baited SELEX, functional group-guided selection	1. Breaks the affinity bottleneck of concentration pressure-based screening. 2. Integrates sulfur(VI) fluoride exchange chemistry and structural constraints, even achieving a 10-fold improvement in affinity. 3. Potential to select a therapeutic aptamer.	1. Discovery of substantial therapeutic aptamers is still limited. 2. Clinical application and therapeutic translation need to be further advanced.
AI-driven aptamer selection platform	1. RaptGen: VAE + HMM 2. AptaDiff: diffusion-based generation 3. DL-SELEX: structure-enhanced library design 4. SILEX: single-round data clustering 5. RhoDesign: structure-to-sequence transformer	1.Breaks the inherent limitations of library diversity (extending exploration spaces from 10^14^ to 10^24^ variants) and experimental bias. 2. Evolves from multiround sequencing data to single-round data-driven models, and finally achieves de novo design (reducing iterations by up to 80%-100%). 3. Possesses the potential for selecting aptamers tailored to specific bioactive sites.	1. Current AI frameworks cannot fully simulate the intricate physiological environments or the complex cell membrane. characteristics 2. The practical efficacy of AI-designed aptamers requires further experimental validation.
Multimodal-integrated aptamer selection platform	1. CRISPR-Hybrid: CRISPR + FACS 2. GRAPE-LM: CRISPR + language model 3. SPARK-seq: full-process integration of CRISPR-based genetic perturbation, single-cell multiomics and AI analysis.	1. Accelerates the target identification within cell-based selection. 2. Enables precise in situ mapping of aptamer–target interactions and binding kinetics. 5. Has the potential to simultaneously discover aptamers and clinically actionable cell-surface targets.	Further in-depth research is required to fully realize its value in disease diagnosis, therapeutics, and clinical transformation.

### High-throughput aptamer selection platform

High-throughput aptamer discovery platforms circumvent the bottlenecks of traditionally labor-intensive centrifugation or magnetic separation with advanced partitioning techniques.^85–88^ Currently, evolutionary selection platforms include microfluidic architectures governed by precise fluid dynamics, hydrogel matrices leveraging differential molecular diffusion, and gradient-based thermodynamic elution. Such high-throughput isolation of target-binding sequences from massive libraries significantly accelerates the selection process and even reduces multiple cycles to a single round.

Within the realm of high-throughput selection, microfluidics-assisted screening has emerged as the most representative technology. Recently, this field has evolved from simple iterative enrichment to single-round quantitative generation of aptamers. Pro-SELEX represents a paradigm shift by integrating efficient particle display with microfluidic sorting and bioinformatics.^81^ As illustrated in Fig. 2a, DNA libraries are converted into monoclonal microspheres via emulsion polymerase chain reaction (PCR) and incubated with magnetic targets. Pro-SELEX employs controlled laminar flow to physically fractionate microspheres based on their magnetic load, enabling the rapid discovery of aptamers with precisely defined affinities to meet diverse application requirements. Furthermore, on the basis of microfluidic sorting, a massively parallel screening^89^ has been designed to convert virtually any aptamer into a binding-induced conformational molecular switch without prior structural knowledge, facilitating the systematic identification of functional probes for real-time detection and targeted drug delivery.

However, microfluidic methods are often constrained by specialized instrumentation and limited library throughput. UltraSelex^90^ is subsequently developed by standardizing the collection of successive wash fractions, which tracks binders from all fractions to identify initially rare sequences. UltraSelex handles much larger RNA libraries (10^13^–10^14^) and demonstrates broader applicability across both small-molecule and macromolecular targets without sophisticated equipment. To further minimize nonspecific binding, the hydrogel for aptamer selection (HAS)^91^ proposes an instrument-free or bead-free SELEX system, utilizing the concentration gradient of free molecules within a three-dimensional (3D) nonfouling macroporous hydrogel to facilitate a coupled diffusion-binding process. Therefore, HAS transcends nonspecific binding limitations and provides potential for selection against living cells.

While a variety of high-throughput technologies have emerged, they lack extended refinement and in-depth mechanistic studies, such as optimizing the stability of these systems, adapting them for complex clinical samples, or transitioning them into commercialization and productization. Moreover, high-throughput screening against living cells continues to be a challenge. Although HAS possesses the theoretical potential to handle the complexity of cellular surfaces, dedicated research against cell-targeted selection is still in its infancy.

### High-affinity aptamer selection platform

Conventional SELEX methodologies primarily depend on concentration pressure, a progressive reduction in target concentration during selection rounds, to enrich for high-affinity sequences.^92,93^ However, this selection pressure is often insufficient to effectively isolate aptamers with high affinity, resulting in a plateau in binding performance.^94^ Consequently, a suite of sophisticated strategies has been successively developed.^24,95^ It includes initial library design with structure-guided epitope targeting or irreversible covalent cross-linking and selection process optimization with nuclease-assisted kinetic filtering. The above approaches are designed to bypass traditional limitations, enabling the discovery of aptamers with high affinity.

Modern platforms integrate latent bioreactive chemistry and structural constraints directly into library design to transcend thermodynamic boundaries. By incorporating electrophilic warheads, proximity-driven reactions can convert transient complexes into permanent covalent conjugates.^96–98^ This enables the rapid discovery of irreversible inhibitors. Alternatively, designing libraries with fixed motif incorporations shifts the selection pressure toward optimizing the local fitness landscape.^99–101^ This ensures that flanking sequences evolve to provide the tertiary interactions necessary to stabilize active conformations and maximize affinity.

Furthermore, functional design has also been introduced to library establishment for either the discovery of high-affinity aptamers or the biological effects of aptamers.^24,102–106^ Strategies such as Blocker-SELEX^107^ and Baited SELEX^108^ use decoy partners or active-site “baits” to isolate aptamers that overlap critical protein‒protein interaction (PPI) interfaces or catalytic pockets. Similarly, functional group-guided selection employs simplified analogs to direct the evolution of anchor motifs against specific chemical groups, overcoming the negative cooperativity of complex molecules to identify binders for undruggable targets.^102^ This evolution toward functional utility is further exemplified by aptamer-based degraders,^109^ which leverage bispecific chimeras to hijack the ubiquitin–proteasome system, converting high-affinity binders into potent therapeutic tools for targeted protein degradation.

Additionally, various DNA-processing enzymes, such as endonuclease^82^ or exonucleases,^110,111^ are incorporated in the selection process to convert “binding events” into “enzymatic resistance” for the generation of high-affinity aptamers.^112^ Specifically, aptamers that undergo a target-induced conformational switch can mask the cleavage site and survive digestion, allowing high-affinity binders with slow dissociation rates to persist in solution while background sequences are rapidly hydrolyzed (Fig. 2b). Therefore, nuclease-assisted strategies act as stringent molecular sieves, ensuring that only sequences with superior structural stability and binding kinetics are retained.

Overall, this transition from screening throughput optimization to sophisticated methodologies based on chemical modification, conformational transitions, or functionalization has significantly increased the discovery of high-affinity aptamers. This evolution provides a solid foundation for the future development of faster and more comprehensive screening platforms. Furthermore, the emphasis on functional discovery promotes the development of aptamers with enhanced biological efficacy, which is vital for accelerating their clinical application and therapeutic impact.

### AI-driven aptamer selection platform

The integration of AI with high-throughput sequencing data has catalyzed a paradigm shift in aptamer discovery, moving from stochastic physical screening to rational computational design.^113–120^ AI-driven platforms leverage advanced machine learning architectures, ranging from generative models to deep reinforcement learning, to decipher the complex nonlinear relationships between sequence, structure, and binding affinity.^121^ By integrating AI into the selection workflow, researchers can quantitatively mine sequence space and predict high-affinity binders from initial selection rounds. These computational strategies circumvent the inherent limitations of library diversity and experimental bias, representing a paradigm shift in functional ligand discovery. The landscape of AI-driven aptamer discovery was fundamentally reshaped in 2022 by the introduction of RaptGen, a pioneering framework that integrated a Variational Autoencoder (VAE) with a profile Hidden Markov Model (HMM) decoder to capture essential motif architectures (Fig. 2c).^83^ Moving beyond traditional computational tools, RaptGen enabled the in silico generation of novel aptamers by navigating a continuous, low-dimensional latent space. However, despite its generative capabilities, the model remained inherently tethered to the sequence-level patterns present in conventional SELEX datasets.

Following the foundational VAE-based architectures, the field has rapidly transitioned toward more sophisticated probabilistic frameworks. By 2024 and 2025, AptaDiff and deep learning-assisted SELEX (DL-SELEX) had substantially expanded this technical repertoire.^117,122^ AptaDiff utilizes diffusion-based generation and motif-dependent embeddings to outperform earlier models in sequence diversity and fidelity. Meanwhile, DL-SELEX optimizes the discovery pipeline by using deep learning to design structure-enhanced initial libraries, reducing experimental iterations by up to 80%. By leveraging unsupervised clustering, the SILEX platform^123^ decodes structure‒function relationships from a single selection round, further bypassing the need for iterative data. Notably, the recently developed RhoDesign platform represents a definitive evolution beyond the RaptGen era by implementing a structure-to-sequence transformer-based architecture.^124^ RhoDesign operates as a structure-driven engine capable of engineering RNA aptamers directly from a target’s 3D point cloud or predefined structural folds, which circumvent the experimental biases and data dependencies inherent in sequence-based models. Therefore, AI-driven platforms have transitioned from models requiring iterative sequencing data to efficient systems such as SILEX that rely on single-round results. Ultimately, this evolution has led to structure-based engines such as RhoDesign, which enable de novo design directly from a target’s structural topology. This shift toward source-design strategies is particularly transformative for functional selection. This allows for the rational engineering of aptamers tailored to specific bioactive sites, thereby enhancing their therapeutic potential.

However, several current limitations in real-world applications severely hinder the translation of these AI-selected designs into viable therapeutics. Most current AI frameworks operate under idealized, rigid-body assumptions that fail to simulate dynamic physiological environments, such as competitive serum protein binding or complex cell-surface matrices. Furthermore, training datasets lack negative, nonbinding sequence data from failed SELEX rounds, which frequently causes generative models to predict candidates with high off-target cross-reactivity. Consequently, transitioning these computational candidates into clinical assets imposes rigorous validation requirements. Validation pipelines must move beyond simple cell-free affinity assays, progressing to comprehensive structural verification to confirm specific binding, accompanied by functional cell-based assays. To balance computational speed with empirical reliability, a pragmatic strategy integrates structure-led algorithms for initial scaffolding with high-throughput experimental feedback loops to iteratively refine the model’s predictive accuracy. Beyond these empirical hurdles, AI-driven platforms face unprecedented regulatory and safety challenges looking toward clinical implementation. Unlike traditional lab-based SELEX, which features a traceable, round-by-round evolutionary trajectory that can be easily audited, deep learning architectures often operate as black boxes. This lack of algorithmic transparency makes it exceptionally difficult to trace the decision-making lineage of a generated sequence, raising strict regulatory concerns regarding reproducibility, batch-to-batch consistency and toxicological predictability. To transform these intelligent computational engines into compliant clinical tools, the field must establish standardized evaluation frameworks, such as minimum data criteria for training sets, open-source benchmarking datasets, and interpretable AI models, to ensure long-term clinical safety and compliance.

### Multimodal-integrated aptamer selection platform

While conventional cell-based selection preserves the native conformation of targets and enhances the practical efficacy of the selected aptamers, it still suffers from a “black box” limitation. The evolution toward multimodal-integrated platforms represents a strategy of comprehensive functional profiling toward aptamers, enabling the resolution of the complex interplay between sequence architecture and biological activity across multiparametric landscapes.

In 2023, the CRISmer system pioneered this transition by repurposing clustered regularly interspaced short palindromic repeats/CRISPR-associated systems (CRISPR/Cas) as an intracellular reporter. By coupling molecular recognition directly to transcriptional outputs, it identifies aptamers against the severe acute respiratory syndrome coronavirus 2 (SARS-CoV-2) spike protein.^125^ This integration of binding events and genetic reporting provides a more biologically authentic selection pressure than traditional methods, ensuring that candidates are prevalidated for their native cellular environments. Furthermore, the CRISPR-Hybrid platform^126^ and the generator of RNA aptamers powered by activity-guided evolution and the language model (GRAPE-LM) framework^127^ introduce deeper layers of multimodal synergy by fusing experimental throughput with predictive intelligence. CRISPR-Hybrid refines the selection process by coupling bacterial hybrid systems with fluorescence-activated cell sorting (FACS), enabling the evolution of high-resolution orthogonal aptamer-RNA-binding protein (RBP) pairs. Complementarily, GRAPE-LM revolutionizes the discovery cycle by integrating CRISPR‒Cas screening data with nucleic acid language models. This synergy allows for the one-round evolution of aptamers, where the model decodes hidden correlations between intracellular performance and sequence motifs to enable data-driven, de novo design.

Notably, the most sophisticated realization of the multimodal paradigm is the recently developed SPARK-seq platform (Fig. 2d), which achieves full-process integration by merging CRISPR-based genetic perturbation with single-cell multiomics.^84^ Unlike standard Cell-SELEX or basic intracellular screening, SPARK-seq provides an all-in-one workflow that concurrently profiles aptamer binding, target expression, and cellular phenotypes at single-cell resolution. This multimodal approach enables the precise mapping of aptamer–target interactions and binding kinetics in situ, facilitating the simultaneous discovery of high-performance aptamers and the identification of clinically actionable cell-surface targets. Therefore, SPARK-seq transforms the selection platform into a multidimensional discovery toolbox for rational aptamer drug development. By fusing CRISPR tools, cell-based screening, multiomics, and AI, multimodal strategies create a unified workflow that unites aptamer selection with target identification and validation. This synergy accelerates aptamer development and clinical translation while offering innovative methodologies for drug target discovery. Further research into these systems will hold profound significance for disease diagnosis, therapeutics, and the elucidation of underlying biological mechanisms.

## New methods for target validation

**Fig. 3:**
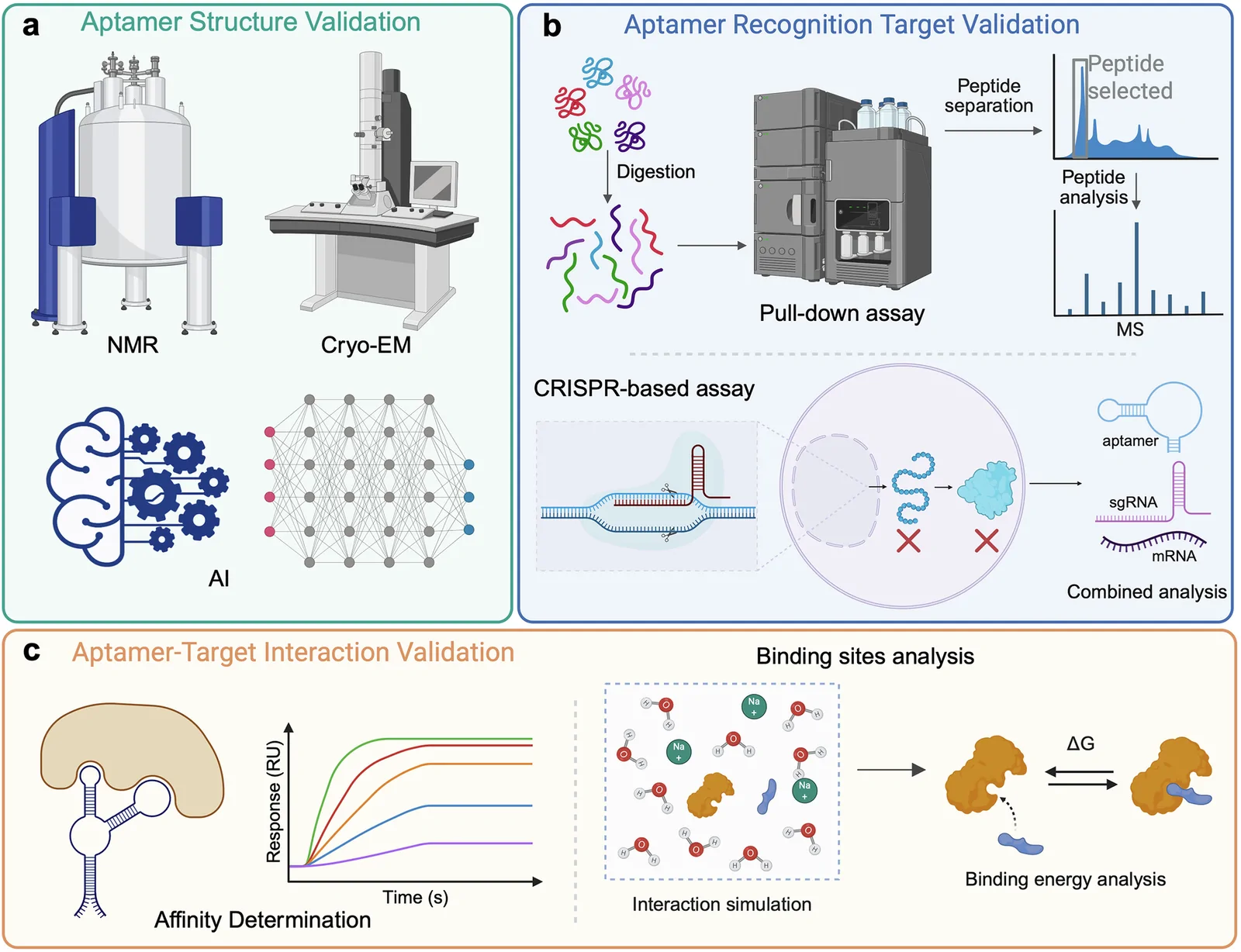
New methods for a target validation framework for the structural and functional characterization of new aptamers. This integrated workflow encompasses three critical stages: **a** aptamer structure validation utilizing high-resolution biophysical tools such as nuclear magnetic resonance (NMR), cryo-electron microscopy (Cryo-EM), and AI-driven predictive modeling to resolve folding architectures; **b** aptamer recognition target validation to confirm target specificity in situ through pull-down assays coupled with liquid chromatography-tandem mass spectrometry (LC‒MS/MS) and CRISPR-mediated functional genomics; and **c** aptamer–target interaction validation, which employs affinity characterization to determine binding constants alongside interaction mapping and deep learning to precisely identify binding sites and interaction patterns. Some resources in this figure come from BioRender

The rapid evolution of screening platforms has effectively addressed the discovery bottleneck, yielding a vast array of potential aptamer candidates. However, the functional translation of these lead aptamers into precision therapeutics or diagnostics necessitates a rigorous, multidimensional validation framework. This validation phase is critical for deciphering the structural rationale and ensuring target specificity within complex physiological environments. In this review, we focus on the emerging methodologies for validating aptamer architectures (Fig. 3a) and identifying targets in situ (Fig. 3b). We also conclude the technologies for affinity and binding site determination toward aptamer–target interactions in this section (Fig. 3c).

### Aptamer structure validation

Aptamer structural characterization commonly relies on low-resolution techniques such as circular dichroism (CD) spectroscopy^128,129^ and thermodynamic folding algorithms (e.g., Mfold^130,131^, NUPACK^132,133^). While these methods provide basic insights into secondary structural motifs (e.g., hairpins), they only offer average data and often fail to predict tertiary folding or specific intermolecular interactions within complex physiological environments. Consequently, they are insufficient for guiding rational drug design or elucidating precise structure-activity relationships. To overcome these limitations, relevant studies have shifted toward atomic-resolution imaging and generative artificial intelligence for rapid, high-accuracy structural prediction (Table 3).

**Table 3:** Comparison of aptamer structure validation methods

Aptamer structure validation	Nuclear magnetic resonance (NMR)	Cryo-electron microscopy (Cryo-EM)	AI-driven prediction (e.g., AlphaFold 3)
Critical function	Determines native solution topology and conformational diversity.	Visualizes large, complex 3D architectures.	Provides rapid, high-fidelity computational 3D modeling.
Throughput	Low (1–2 sequences/month). Limited by sequential sample preparation and manual/complex spectral assignment.	Low to Medium (1–4 sequences/week). Bottlenecked by grid preparation optimization and intensive computation for 3D reconstruction.	High (>100 sequences/day). Capable of high-throughput screening of massive aptamer sequence libraries simultaneously
Sample requirements	Requires small-to-medium DNA/RNA sequences in solution.	Captures aptamers in a native, frozen-hydrated state using vitrification.	No physical sample needed.
Cost	Moderate ($ 500-200/sample). Instrumental runtime cost and stable isotope labeling cost.	Very High ($ 2000–4000/day, instrument fee). Expensive capital instrumentation, specialized preparation, and intensive high-performance computing fees.	Low (<$ 0.1/prediction). Standard computing hardware or scalable cloud-computing credits for GPU access.
Time	Weeks to Months. Requires multidimensional data collection and manual peak assignment and structure calculations.	Days to Weeks. Requires hours to days for data collection, followed by intensive 2D classification and 3D refinement.	Minutes to hours. Prediction is typically completed within minutes to a few hours per target, depending on model parameters and sequence lengths.
Key advantages	Captures dynamic equilibrium and adaptive binding in near-physiological liquid environments.	Bypasses molecular weight ceilings and isotopic labeling.	Serves as a rapid structural filter for sequences and provides confidence metrics.
Main limitation	Limited by molecular weight constraints and the need for intensive isotopic labeling.	Historically difficult for small targets (often requires DNA origami templates).	Results are predictive benchmarks; they still require experimental verification for final proof.
Specific example	Sgc8c Aptamer: Resolved the intricate 3D fold to provide a blueprint for functional truncation.^141^	TBA Aptamer: Visualized as a guest molecule within a rigid DNA origami framework to resolve its small 8 kDa structure.^150^	G-quadruplexes: Accurately predict noncanonical folding patterns and binding interfaces.^155^

#### Nuclear Magnetic Resonance (NMR) Spectroscopy

NMR is uniquely capable of capturing the dynamic equilibrium and adaptive binding transitions, enabling the determination of the conformational diversity of small-to-medium DNA/RNA aptamers in near-physiological liquid environments. Specifically, NMR can definitively assign the glycosidic bond angles (syn vs. anti) and hydrogen-bonding patterns (Hoogsteen vs. Watson-Crick) that define the aptamer’s unique folding.^134–138^

Traditionally, aptamer structure studies rely on highly complex NMR analysis. For instance, Xu et al. used NMR to reveal that the aflatoxin B1 (AFB1) aptamer (AF26) forms bulges and hairpins via the GC base pair, GGC triple and multiple T bases to closely bind to AFB1.^139^ Similarly, Jiang et al. determined the complex structure of adenosine triphosphate (ATP) and binding aptamer (1301b_v1),^140^ uncovering a distinctive L-shaped architecture and highlighting an adaptive binding mechanism where the aptamer transitions from a semifolded state to a stable complex upon ligand intercalation. To evolve the NMR analysis process, He et al. first proposed a simplified NMR strategy by combining free-state structural determination with site-specific perturbation mapping.^141^ They successfully resolved the 3D fold of the 41-nt sgc8c aptamer, revealing an intricate three-way junction (3WJ) stabilized by long-range tertiary interactions, such as the trans-space hydrogen bonding between C7 and G17. This streamlined strategy efficiently uncovers the structural basis for target recognition and provides a clear template for enhancing the stability and affinity of nucleic acids. Compared with other structure determination techniques for aptamers, NMR is more accessible for laboratory operation. However, current NMR requires large sample quantities and high technical expertise, leading to high characterization costs. Therefore, improvements in NMR instrumentation or the development of simplified analytical strategies could more effectively serve the advancement of therapeutic aptamer-based drugs.

#### Cryo-electron microscopy (Cryo-EM)

Bypassing the resolution ceilings and the intensive isotopic labeling requirements, cryo-EM provides in-depth structural insights that transcend NMR or small-angle X-ray scattering (SAXS) methods.^142^ Cryo-EM^143–146^ mainly captures aptamers in their native, frozen-hydrated state to directly visualize the phosphate backbone and base-stacking interactions, providing high-precision and structure-guided science. Specifically, cryo-EM has been instrumental in resolving the XenoAptamer,^147^ where it revealed how hydrophobic Ds-Px base pairs create a stable tertiary fold that allows the aptamer to distinguish between subtle amino acid variations in viral variants. Similarly, by utilizing a group II intron scaffold, researchers successfully determined the 2.5 Å resolution structure of the thiamine pyrophosphate (TPP) riboswitch aptamer,^148^ capturing its open Y-shaped conformation in the apo state and providing a high-definition map of its ligand-binding pocket. Beyond structural mapping, cryo-EM also offers deep insights into functional mechanisms. For example, cryo-EM analysis of the insulin receptor trapped by the A62 agonist aptamer^149^ demonstrated that the aptamer mimics natural insulin coordination to selectively activate metabolic signaling. Despite the challenges of imaging small molecules, the implementation of rigid DNA origami templates as alignment frameworks has enabled cryo-EM to successfully visualize the small 8 kDa thrombin-binding aptamer (TBA), showcasing the versatility of cryo-EM in resolving low-molecular-weight targets within a programmable structural context.^150^

Collectively, cryo-EM has revolutionized our understanding of aptamer architectures, while the integration of DNA origami templates has bypassed the resolution limits in imaging small molecules. However, such rigid frameworks may constrain the inherent flexibility of aptamers, potentially resulting in structural models that deviate from their free states. Additionally, the high cost of Cryo-EM instrumentation also limits its accessibility compared to NMR. Consequently, NMR remains the mainstream laboratory technique for target identification, whereas establishing cryo-EM as a universal tool for routine aptamer characterization is a big challenge.

#### AI-driven structural prediction

AI algorithms can accurately predict nucleic acid folding patterns,^151,152^ including noncanonical elements such as G-quadruplexes and pseudoknots.^114,153^ This capability allows for the precise mapping of structural features and binding interfaces,^154^ facilitating the rational design of nucleic acid-based affinity reagents with high structural fidelity. A prominent example of this advancement is AlphaFold 3,^155^ which has expanded its predictive power to include nucleic acids and small-molecule targets, offering a direct 3D modeling approach for aptamer sequences. Research demonstrates that AlphaFold 3 can effectively recover native conformations, achieving an average backbone root mean square deviation (RMSD) of 1.45 Å for structures within its training set and maintaining high accuracy for many novel sequences. The integration of localized confidence metrics, specifically predicted local distance difference template (pLDDT) and predicted template modeling score (pTM), enables AlphaFold 3 to provide a high-fidelity validation of aptamer conformations, offering a reliable structural benchmark even when empirical evidence is severely limited.

Therefore, AI technology acts as a rapid structural filter, enabling the assessment of the predicted stability and spatial compactness of aptamer sequences. By providing these insights prior to experimental structural biology, it effectively bridges sequence selection with structural validation. However, the inherent flexibility of aptamers causes a discrepancy between AI-generated predictions and the actual conformational dynamics in a solvent. Similar to AI-driven selection development, the incorporation of multiparametric data that mimic physiological conditions will be essential for AI to achieve high-fidelity structural analysis of aptamers in their functional states. Furthermore, the clinical translation of computationally predicted structures faces similar regulatory hurdles due to the lack of unified evaluation standards. Standardizing these predictive methodologies and integrating them into a systematized workflow is highly advantageous for regulatory auditing. Ultimately, developing systematized and harmonized structural assessment frameworks is essential for computationally guided designs to satisfy clinical pharmacology and global regulatory requirements.

### Aptamer recognition target validation

While cell-based selection offers the distinct advantage of selecting aptamers against proteins in their native, physiological conformation on the cell surface, it inherently creates a “black box” problem. Unlike protein-based selection, where the target is known, aptamers generated via cell-based selection bind to specific but unknown molecular features on the target cells.^156–158^ The lack of target identity poses a significant bottleneck for downstream applications. Therefore, target identification and subsequent validation are critical for evolving a raw sequence into a functional biomedical tool.

Typically, the identification of unknown targets is fundamentally driven by affinity-based biochemical strategies. For example, Lv and colleagues utilized stable isotope labeling by amino acids in cell culture (SILAC),^159^ where target cells were grown in “heavy” or “light” isotopic media. The enriched pool and a control library were incubated with these labeled cells, followed by in situ cross-linking and streptavidin-bead purification of the DNA‒protein complexes. Subsequent LC‒MS/MS analysis identified receptor-type tyrosine-protein phosphatase F (PTPRF) as the primary target, showing a significant abundance ratio (over 10-fold) compared to the control. While this pull-down approach is feasible, the reliance on complex lysis and multistep purification poses significant challenges for the detection of low-abundance membrane proteins.

To address these limitations, the SPARK-seq platform recently represents a paradigm shift toward “Aptomics”.^84,160^ By integrating CRISPR-based genetic perturbation with single-cell multiomics, SPARK-seq enables the simultaneous identification of aptamers and their targets in situ without the need for cell lysis or physical enrichment. This high-throughput platform employs a digital deconvolution logic by correlating the loss of aptamer binding with specific protein knockouts across thousands of single cells. SPARK-seq maps thousands of sequences to their targets with unprecedented scale. Such an approach provides a direct, sequence-based map of the aptamer–target landscape, effectively solving the “black box” problem by identifying both the binder and its target in a single integrated workflow. The emergence of SPARK-seq indicates that target identification can be solved through intelligent and integrated technology. This also demonstrates the significant potential of SPARK-seq as a target discovery tool for pharmaceutical development and clinical therapy. Furthermore, it remains both promising and challenging to explore whether this strategy can be extended to tissue-based or in vivo aptamer selection and target identification platforms. Nevertheless, such an advancement would substantially facilitate the clinical translation of aptamers.

### Aptamer–target interaction validation

Following the identification of a candidate biomarker, a rigorous validation of the aptamer–target interaction is indispensable to confirm binding specificity, quantify affinity parameters, and elucidate the molecular basis of recognition.^161–163^ This multidimensional verification process transitions from fundamental biophysical characterization to high-resolution and functional assessment in complex biological environments.

#### Characterization of aptamer binding affinity and kinetics

**Fig. 4:**
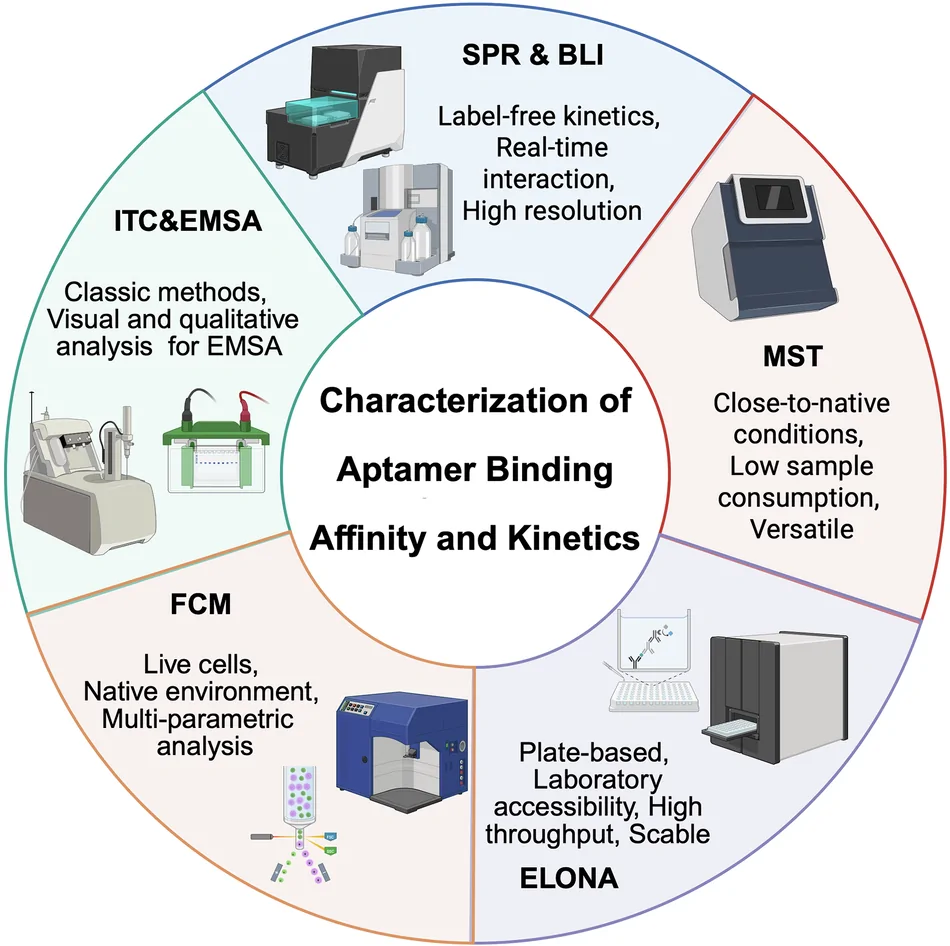
Scheme of diverse characterization strategies of aptamer binding affinity and kinetics. Isothermal titration calorimetry (ITC) and electrophoretic mobility shift assays (EMSAs) serve as classical tools for thermodynamic profiling and qualitative complex assessment. Surface plasmon resonance (SPR) and biolayer interferometry (BLI) represent the transition toward high-resolution, label-free, and real-time biophysical characterization. Microscale thermophoresis (MST) provides a versatile, solution-phase alternative for measurements in close-to-native conditions with minimal sample consumption. For broader laboratory accessibility, the enzyme-linked oligonucleotide assay (ELONA) offers a scalable, plate-based platform for high-throughput screening. Finally, flow cytometry (FCM) enables the determination of affinity directly on live cells, ensuring that the aptamer’s performance is validated within its native physiological environment. Some resources in this figure come from BioRender

The determination of binding affinity and kinetic parameters constitutes the primary benchmark for evaluating an aptamer’s potency, driving the continuous development of diverse analytical technologies (Fig. 4). Traditionally, isothermal titration calorimetry (ITC)^17^ provides a complete thermodynamic profile, and electrophoretic mobility shift assays (EMSAs) offer a straightforward qualitative assessment of complex formation, but both are limited by low-resolution or low-throughput characterization.

Modern analytical platforms such as SPR^164^ and BLI^165^ have become essential for providing high-resolution and kinetic data. SPR relies on the measurement of refractive index changes near a gold-coated sensor surface to track molecular interactions with extreme sensitivity, enabling the calculation of precise kinetic constants. Similarly, BLI utilizes white-light interferometry to monitor molecular thickness changes on a biosensor tip, offering a high-throughput alternative that is particularly robust when handling crude or refractive samples. For broader laboratory accessibility and high-throughput screening, the enzyme-linked oligonucleotide assay (ELONA)^166–169^ serves as a powerful plate-based platform. As an aptamer-based analog of enzyme-linked immunosorbent assay (ELISA), ELONA leverages the high specificity of aptamers to capture or detect targets, producing colorimetric, fluorescent, or chemiluminescent signals. This method is particularly valued for its scalability, allowing for the simultaneous Kd determination of multiple aptamer candidates in a standardized format.

Complementing these surface-immobilization techniques, microscale thermophoresis (MST)^170,171^ has gained prominence for its ability to measure interactions in close-to-native, solution-phase conditions. By detecting the directed movement of molecules along a microscopic temperature gradient, MST allows for the determination of dissociation constant *K*_*d*_ values with minimal sample consumption. Its major strength lies in its versatility, as it remains highly effective in complex biological matrices such as cell lysates or blood serum. To further ensure that the aptamer maintains its performance in a complex physiological context, FCM-based *K*_*d*_ analysis is performed directly on live cells,^172–175^ offering a more biologically relevant validation than traditional cell-free assays. By incubating target-expressing cells with a concentration gradient of fluorescently labeled aptamers, the apparent *K*_*d*_ can be calculated from the resulting saturation binding curves based on the mean fluorescence intensity (MFI). FCM can evaluate the aptamer’s binding behavior in its native environment, accounting for critical cellular factors such as target protein density, posttranslational modifications, steric hindrance from the glycocalyx, and membrane fluidity.

Collectively, aptamer affinity characterization frameworks reflect a deepening development from the measurement of isolated physical constants to the simulation of complex biological environments. While classical tools such as ITC and EMSA laid the foundation for thermodynamic profiling and qualitative assessment, the adoption of real-time kinetic technologies (SPR and BLI) has enabled researchers to gain critical insights into the contribution of association rate constant *k*_*on*_ and dissociation rate constant *k*_*off*_ to biological efficacy. Notably, as an aptamer-based analog of ELISA, ELONA not only facilitates the parallel affinity validation of numerous candidate sequences but also demonstrates that aptamers can be seamlessly integrated into existing clinical diagnostic tools. The ultimate trajectory of characterization strategies lies in the enhancement of environmental fidelity. The robust performance of MST in complex matrices (such as serum or lysates), alongside the application of FCM on live cell surfaces, effectively bridges the gap between in vitro assays and in vivo environments. In particular, FCM provides a more comprehensive evaluation by accounting for dynamic factors such as membrane fluidity and the native conformational states of target proteins. Furthermore, the characterization of affinity is gradually evolving to incorporate into the aptamer selection process for simplified and integrated development in the future.

#### Analysis of aptamer binding sites

As previously summarized, NMR and cryo-EM have served as indispensable cornerstones for elucidating the precise spatial configurations and atomic-level interactions governing aptamer–target recognition. Nevertheless, this field is currently undergoing a transformative shift toward a data-driven, AI-integrated paradigm. The emergence of diverse AI methodologies has revolutionized the analysis of binding sites and molecular interactions between aptamers and their targets, marking a clear evolutionary trajectory to sophisticated deep learning architectures.^176,177^

Earlier computational frameworks, such as the protein–aptamer interaction prediction web server (PPAI) (integrating AdaBoost and Random Forest)^178^ and aptamer–protein interaction prediction (APIPred) (utilizing XGBoost)^179^, established the foundation of this field by leveraging intricate sequence-derived features, such as k-mer frequencies for aptamers and pseudoamino acid composition (PseAAC) for proteins, to predict binding affinities with high precision. Building upon these methodologies, AptaNet introduced deep neural networks to integrate multidimensional features from both partners, significantly enhancing the identification of key interaction interfaces. Most recently, the paradigm has been developed into a transformative model such as AptaTrans,^180^ which employs transformer-based encoders to capture monomer-level representations and global contextual information.

Furthermore, the integration of large language models (LLMs) leads to aptamer evaluation, exemplified by the development of RaptScore.^181^ Moving beyond traditional metrics often constrained by sequence length or library diversity, RaptScore utilizes an LLM-based algorithm to evaluate the binding activity of arbitrary sequences by decoding the underlying language of aptamer–target recognition. This allows for versatile, cross-platform evaluation across diverse SELEX datasets, empowering researchers to perform in silico truncation and lead optimization without compromising binding efficiency. By capturing the global contextual dependencies of nucleotide sequences, LLM-based tools offer a robust and scalable solution for navigating vast sequence spaces to identify high-affinity candidates.

The practical validation of these AI-derived insights is often facilitated by competitive binding assays and finalized through site-directed mutagenesis.^141,182,183^ Through systematic mutations on the protein or specific nucleotide substitutions on the aptamer, researchers can pinpoint the exact residues or bases required for the interaction, thereby confirming the computational predictions. By synergizing the advanced computational predictions with targeted experimental validation, the field is poised to accelerate the functional development and clinical translation of next-generation aptamer therapeutics.

## New methods for molecular diagnostics

Aptamers have antibody-like target recognition and distinct advantages, including facile selection, low-cost synthesis, versatile chemical modification, and high physicochemical stability. They have emerged as powerful molecular recognition elements for biomedical diagnostics.^76,184^ Since their discovery, extensive efforts have driven the development of diverse aptamer-based detection strategies.^10,185^ The convergence of DNA nanotechnology, advanced signal amplification mechanisms, and nanomaterial-enabled platforms has further accelerated the evolution of innovative diagnostic systems,^19,20^ some of which have achieved clinical validation and commercial translation.^186^ More recently, escalating demands for rapid, multiplexed, and high-sensitivity analysis, together with continuous technological advances, have propelled the emergence of next-generation aptamer-based platforms, positioning them as key enablers of precision diagnostics. In this section, we highlight representative recent advances in molecular diagnostic methodologies.

### Mechanism of aptamers in molecular diagnostics

**Fig. 5:**
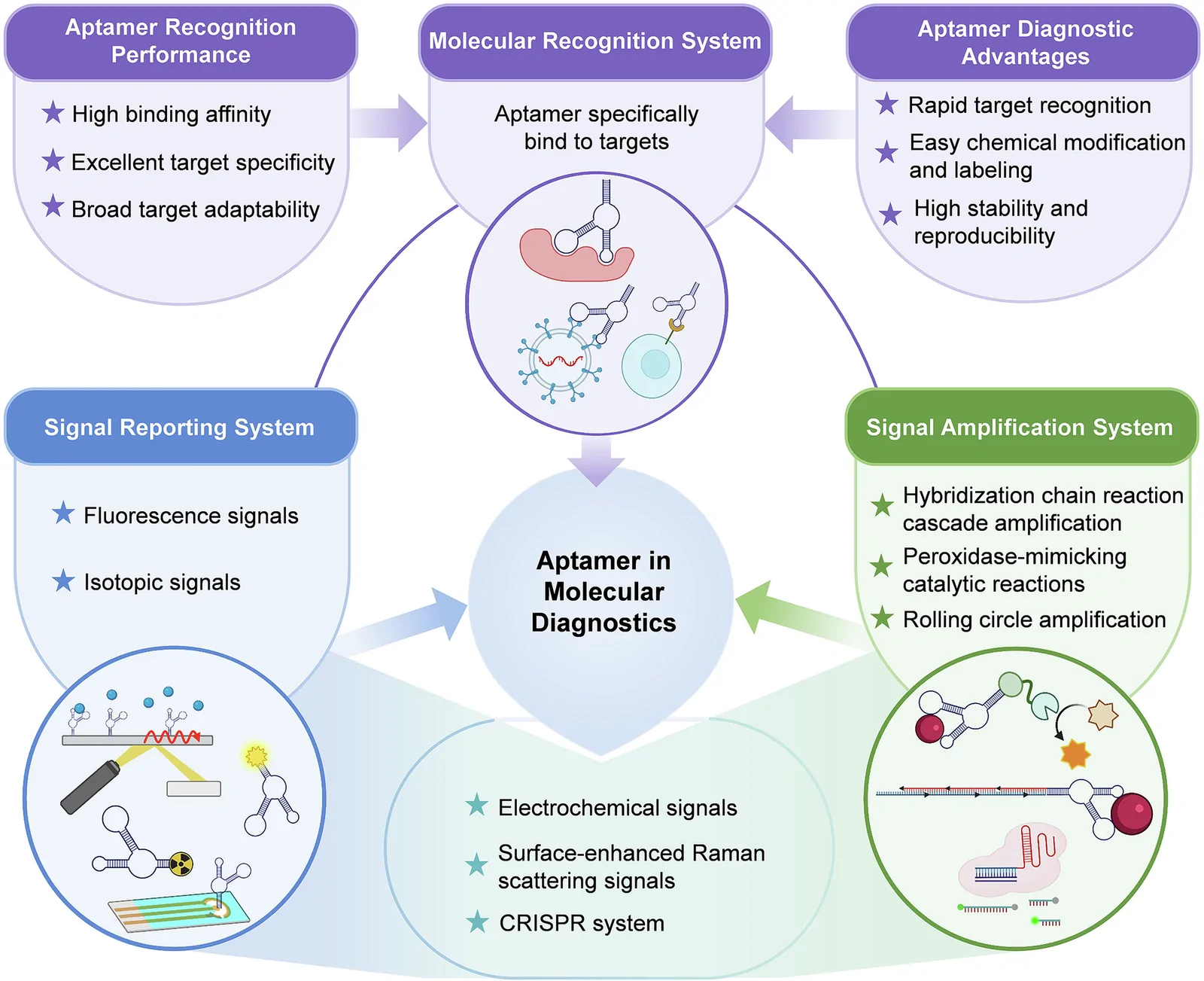
Schematic illustration of the key components constituting an aptamer-based detection system for molecular diagnostics. This figure depicts the three integrated modules of aptamer-based diagnostic platforms: the molecular recognition system, employing target-specific aptamers or their derivatives for selective analyte binding; the signal reporting system, transducing recognition events into measurable outputs such as fluorescence, electrochemical, or SERS signals; and the signal amplification system, utilizing strategies like HCR, RCA, peroxidase-mimicking catalysis, or CRISPR-based systems to enhance sensitivity. Notably, the reporting and amplification systems often overlap, with amplification strategies directly augmenting signal outputs. Some resources in this figure come from BioRender

Aptamer-based molecular diagnostic platforms typically consist of three integrated modules: molecular recognition, signal reporting, and signal amplification (Fig. 5).^10,19,185^ The recognition module relies on target-specific aptamers or their derivatives, which selectively bind analytes through in vitro selection. Upon target binding, this interaction is transduced into measurable outputs by the reporting module, including fluorescence, isotopic, electrochemical, or surface-enhanced Raman scattering (SERS) signals.^20^ To overcome low target abundance and weak signal intensity, amplification strategies, such as hybridization chain reaction (HCR), rolling circle amplification, peroxidase-mimicking catalysis, and CRISPR-based systems, are often incorporated.^10,184,185,187^ Notably, signal reporting and amplification functions are frequently coupled rather than strictly separated. Through rational integration of these modules and validation in complex biological matrices, robust diagnostic systems can be established. With advances in disease biology and technology, molecular diagnostics are evolving toward rapid, noninvasive, real-time, and multiplexed detection with ultrahigh sensitivity and specificity. Concurrently, technological priorities include lowering detection limits, enhancing anti-interference capability, enabling automation and system integration, and incorporating digitalization and artificial intelligence while maintaining cost-effectiveness and accessibility.^184^ Cancer diagnostics remain the primary focus, alongside emerging needs in infectious disease response and chronic disease monitoring,^20^ collectively driving the development of next-generation molecular diagnostic platforms.

### New aptamer-based detection strategies

**Fig. 6:**
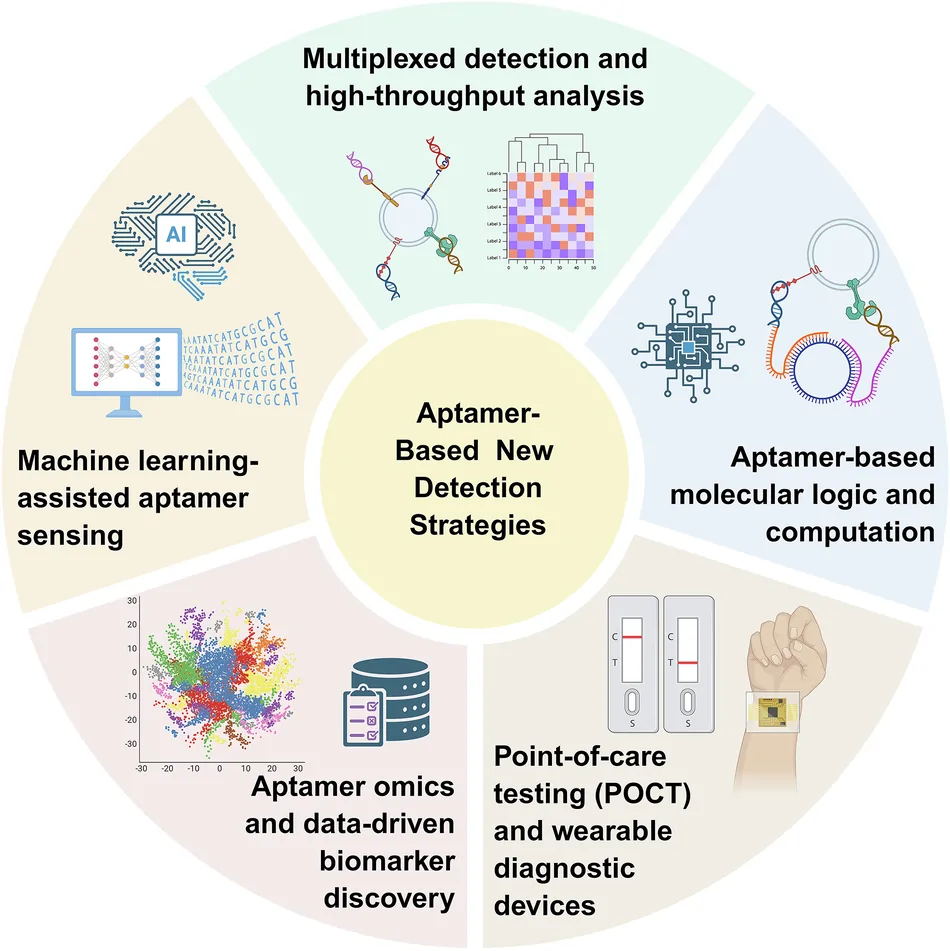
Schematic illustration of representative aptamer-based detection strategies. This figure showcases five advanced aptamer-based detection strategies: (1) multiplexed detection and high-throughput analysis, employing aptamer panels with microarrays, microfluidics, or mass cytometry for parallel biomarker profiling; (2) aptamer-based molecular logic and computation, utilizing DNA circuits and Boolean operations for precise biomarker-integrated recognition; (3) machine learning-assisted aptamer sensing, leveraging AI models for data decoding and assay optimization; (4) aptamer omics and data-driven biomarker discovery, enabling high-throughput, sequencing-based mapping of aptamer–target interactions; (5) POCT testing and wearable diagnostic devices, integrating aptamers into flexible sensors for continuous, real-time, and minimally invasive monitoring. Some resources in this figure come from BioRender

In response to the challenges faced at both the clinical and technological levels, extensive research has been conducted on aptamers to address the essential needs of molecular diagnostics (Fig. 6). These efforts include the development of multivalent and high-throughput aptamer-based detection methods, rapid and integrated molecular logic operations, large-scale data analysis and machine learning based on aptamer-derived datasets, innovative aptamer omics (aptomics) approaches for comprehensive data analysis, and advancements in automated, integrated point-of-care testing (POCT) and wearable device technologies.^84,188–192^ This section will review recent representative achievements, systematically summarize the latest technological advancements, and provide guidance for designing novel molecular diagnostic platforms. Table 4 summarizes the emerging aptamer-based detection strategies.

**Table 4:** Summary of emerging aptamer-based detection strategies

Strategy category	Key technologies	Representative advances	Key advantages	Quantitative performance	Clinical translation stage
**Multiplexed detection and high-throughput analysis**	Multiple aptamer panels; Microarrays, microfluidics, mass cytometry; NGS	Simultaneous detection of PD-L1, CD71, EpCAM, and CA19-9 on sEVs for colorectal cancer diagnosis^196^; ProteoFish-SELEX strategy to screen cancer-specific aptamer combinations from clinical^189^; Metal-labeled aptamers combined with mass cytometry for single-cell proteomic profiling^203^	Enhanced sensitivity and specificity; reveals disease heterogeneity and molecular subtyping; improves diagnostic efficiency	Sensitivity: 92%; Specificity: 86.7%; Accuracy: 90% (CRC diagnosis)^196^; AUC > 0.96 for multicancer discrimination^189^; Single-cell resolution with >40 parameters^203^	Clinical validation (CRC, OV, LC cohorts)^189,196^; Research use (single-cell proteomics)^203^
**Aptamer-based molecular logic and computation**	DNA molecular logic circuits; RCA; HCR	Synchronous DNA logic circuit using ATP and PTK7 aptamers for tumor recognition^208^; Aptamer-triggered DNA assembly anchoring gold nanoparticles onto CTCs for colorimetric readout^209^; RCA-based amplification of CD33/CD123 coexpression signals to identify leukemia stem cells^210^	Instrument-free visual readout; low detection limit; ultrahigh specificity and sensitivity	LOD: 4 cells/mL (CTCs)^209^; LOD: <10 cells/mL (LSCs)^210^; K_*d*_: 3.24 nM (ATP-dependent)^208^	Preclinical (in vitro/in vivo models)
**Machine learning-assisted aptamer sensing**	Machine learning models; Bayesian optimization; Fluorescence barcoding flow cytometry	Decoding information from six aptamers and size-encoded microbeads for hepatocellular carcinoma diagnosis^214^; XGBoost model to optimize polymerization and extraction conditions^191^	Enhanced diagnostic efficiency; global condition optimization; reduced cost and time	Diagnostic accuracy: 94.12% (HCC, AUC 99.3%)^214^; 22.2% reduction in cross-linker use, 57% shorter polymerization time^191^	Clinical cohort validation (84 samples)^214^; Research optimization
**Aptomics**	High-throughput sequencing; SPARK-seq platform	SPARK-seq integrating CRISPR perturbation with single-cell sequencing to identify >5500 aptamer sequences^84^	Single-cell resolution protein quantification; systematic mapping of aptamer–target interactions; scalable specificity decoding	5535 aptamers mapped to 8 surface proteins; PTK7 prediction accuracy ~97%^84^	Research (high-throughput discovery platform)
**POCT and wearable diagnostic devices**	Differential circuit design; Electrochemical aptamer-based sensors; Flexible OFETs	Differential circuit enabling drift-free signals in OFET sensors under stretching and temperature variation^192^; Electrochemical aptamer-based sensor patch for continuous, accurate monitoring of drug concentrations in interstitial fluid^42^	Strong anti-interference capability; high engineering reliability; clinically feasible human-level monitoring	Continuous monitoring (drug concentration); Drift-free signal under mechanical strain^192^	Clinical pilot (human volunteer study)^42^; Advanced prototype^192^

#### Multiplexed detection and high-throughput analysis

Although early studies largely focused on single aptamers targeting individual proteins, increasing recognition of disease heterogeneity has highlighted the limitations of such approaches for accurate diagnosis. This has driven a shift toward multitarget detection strategies. In recent years, aptamer-based diagnostic platforms have increasingly incorporated multiple aptamers to enable simultaneous profiling of protein biomarker panels, representing a key trend in clinical molecular diagnostics.^185,188,190^ Early efforts have explored the development of multiplexed aptamer-based platforms for the simultaneous detection of multiple protein targets to address the growing demand for comprehensive biomarker analysis.^193–195^ Early multiplexed systems demonstrated the feasibility of concurrently detecting biomarkers such as programmed death-ligand 1 (PD-L1), cluster of differentiation 71 (CD71), epithelial cell adhesion molecule (EpCAM), and carbohydrate antigen 19-9 (CA19-9) on small extracellular vesicles (sEVs) for colorectal cancer diagnosis, achieving high sensitivity and accuracy in clinical validation.^196^ Subsequent advances have further expanded target coverage, integrating larger aptamer panels against cancer-associated membrane proteins (e.g., cluster of differentiation 63 (CD63), prostate-specific membrane antigen (PSMA), hepatocyte growth factor receptor (c-Met), PD-L1, EpCAM, mucin 1 (MUC1), protein tyrosine kinase 7 (PTK7), human epidermal growth factor receptor 2 (HER2), and carcinoembryonic antigen (CEA) with amplification strategies such as hybridization chain reaction (HCR) and cyclic signal regeneration, enabling high-dimensional and iterative analysis within single samples.^197^ Collectively, multiplex aptamer-based detection not only enhances sensitivity and specificity through integrated molecular readouts but also provides critical insights into disease heterogeneity and molecular subtyping, underscoring its growing importance in precision diagnostics.

With the rapid expansion of multiplexed information, throughput has emerged as a key bottleneck in multiaptamer molecular diagnostics. To address this challenge, high-throughput technologies have been increasingly integrated with aptamer-based systems, enabling parallel analysis of large numbers of targets and samples. Platforms such as microarrays,^198–200^ microfluidics,^201,202^ and mass cytometry^203,204^ have substantially improved diagnostic efficiency. For example, microarray-based solid-state nanopore arrays functionalized with specific aptamers enable femtomolar-level detection of biomarkers (e.g., alpha-fetoprotein) through parallel signal acquisition and statistical analysis.^198^ Further advances have coupled aptamers with next-generation sequencing (NGS), exemplified by the ProteoFish-SELEX strategy. This leverages nanoparticle-protein corona interactions to directly screen cancer-specific aptamer combinations from clinical serum. This method enables high-throughput and multiplexed discrimination of multiple cancer types using hundreds to thousands of aptamers in a single assay.^189^ Despite these advances, the increasing system complexity associated with high-throughput approaches poses challenges for clinical translation. In this context, mass cytometry offers a promising alternative by combining metal isotope labeling with time-of-flight mass spectrometry, allowing simultaneous measurement of dozens of parameters without spectral overlap. By substituting antibodies with metal-labeled aptamers, this platform enables multiplexed, high-throughput single-cell proteomic profiling while maintaining a relatively streamlined assay design.^203^

#### Aptamer-based molecular logic and computation

Although multiplexed and high-throughput aptamer-based platforms offer powerful tools for profiling heterogeneous tumors, their practical application is often constrained by complexity, cost, and limited suitability for rapid and user-friendly detection. To address these challenges, aptamer-based molecular logic and computation strategies have recently emerged as a promising alternative.^190,205,206^ Aptamer-based molecular logic and computation strategies have drawn conceptual inspiration from the adaptive immune response simulator developed by Han et al.^207^, a DNA reaction network that mimics immune recognition, amplification, and memory through cascaded strand displacement. Based on this design strategy, a synchronous DNA molecular logic circuit using ATP and PTK7 aptamers was constructed, allowing selective recognition of high-PTK7-expressing tumors under the weakly acidic and ATP-rich conditions of the tumor microenvironment.^208^ Although this strategy initially achieved molecular logic and computation, its reporting system remains relatively complex for clinical applications. Subsequent advances have focused on simplifying readouts and improving clinical applicability. For instance, a strategy was designed to anchor catalytic gold nanoparticles onto the surface of circulating tumor cells (CTCs) via aptamer-triggered DNA assembly, generating 3,3′,5,5′-tetramethylbenzidine (TMB) colorimetric signals. This method employs multiple aptamers to recognize CTCs and integrates DNA logic devices constructed through a hybridization chain reaction, enabling precise one-step detection of CTCs. It achieves a detection limit of 4 cells/mL and supports instrument-free visual readout, providing a simple and cost-effective approach for cancer recurrence monitoring based on circulating tumor cells.^209^ Building on these molecular logic and computation-based detection strategies, rolling circle amplification (RCA) was explored to enhance both the reporting and signal amplification systems. By employing RCA to amplify signals triggered by the coexpression of cluster of differentiation 33 (CD33) and cluster of differentiation 123 (CD123) on the surface of leukemia stem cells, this approach ensures ultrasensitive and highly specific phenotypic recognition.^210^ To achieve higher specificity and sensitivity and to enable the precise identification and integrated interpretation of multiple biomarkers in complex biological samples, more advanced aptamer-based DNA logic detection circuits have been developed. By executing Boolean logic operations, such as “AND”, “OR”, and “NOT”, these circuits enable accurate computation and integration of protein signals on the surface of extracellular vesicles (EVs).^211^ Upon fusion, encapsulated RNA walkers are activated by target exosomal microRNAs (miRNAs) to trigger amplified fluorescence signals, enabling highly accurate and sensitive detection of exosomal miRNAs in clinical samples.

#### Machine learning-assisted aptamer sensing

The development of multitarget aptamer analysis and logic-gated molecular computation has greatly enhanced diagnostic efficiency while simultaneously generating a substantial increase in molecular diagnostic data. Traditional manual analysis methods have become a bottleneck for handling such large datasets, highlighting the critical importance of developing intelligent, machine learning-assisted aptamer sensing tools.^212,213^ Traditional optimization typically relies on single-factor experiments, where parameters are adjusted individually. However, this approach is time-consuming and labor-intensive, and it fails to capture interactions between multiple parameters or ensure globally optimal conditions. In an aptamer-labeled nanoparticle-protein corona-based fluorescent barcoding flow cytometry platform, machine learning-assisted data analysis was used to decode the combined information from six aptamers, size-encoded fluorescent microbeads, and flow cytometry, enabling multiplexed analysis of protein biomarkers for hepatocellular carcinoma diagnosis. This approach significantly improved tumor diagnostic efficiency and enabled effective classification of complex protein corona signatures.^214^ In addition to analyzing detection data, machine learning can also optimize aptamer-based assays. Building on results from aptamer detection systems, researchers sought to further improve assay performance by applying machine learning for system optimization. They systematically compared six models—XGBoost, random forest, gradient boosting decision tree (GBDT), AdaBoost, support vector machine (SVM), and classification and regression tree (CAR-T)—to optimize both polymerization conditions (aptamer dosage, cross-linker amount, polymerization time) and extraction parameters (buffer type, Mg^2^⁺ concentration, extraction time, elution time). Bayesian optimization was employed for hyperparameter tuning, with model performance evaluated through cross-validation. XGBoost emerged as the optimal model for condition prediction, ultimately achieving a 22.2% reduction in cross-linker consumption and a 57% shorter polymerization time, demonstrating clear practical value for improving experimental efficiency and reducing costs.^191^ Collectively, these advances highlight machine learning as a critical enabler for the next generation of intelligent, high-performance aptamer-based diagnostic platforms.

#### Aptamer omics and data-driven biomarker discovery

With the increasing demand for multiparameter and multiscale analyses in clinical diagnostics, aptomics has emerged as a powerful technological paradigm. First proposed by Tan et al.,^84,215^ this high-throughput sequencing-based strategy utilizes extensive libraries of randomized DNA fragments to comprehensively profile cell-surface molecules by sequencing bound aptamers. By enabling simultaneous protein quantification at single-cell resolution, aptomics provides a data-driven framework for unbiased biomarker discovery and precision diagnostics, with demonstrated capability in resolving tumor heterogeneity and tracking dynamic molecular changes in complex biological systems. Building on this concept, Luo et al.^84^ introduced SPARK-seq, a high-throughput platform that integrates CRISPR-based genetic perturbation with single-cell sequencing to systematically map aptamer–target interactions in their native cellular context. By linking aptamer binding profiles to gene knockouts, SPARK-seq enabled the identification of over 5500 aptamer sequences against eight distinct surface proteins, highlighting its potential for multiplexed, data-driven discovery. Furthermore, researchers screen aptamers using *k*_*0*__*ff*_ parameters to isolate binders with slow off-rates, which are essential for achieving the stable binding required in diagnostics. When coupled with the deep learning framework SPARTA, this approach establishes a scalable foundation for the aptomics platform. It enables large-scale, sequencing-driven decoding of aptamer specificities and supports the rational design of variants with optimized kinetic properties for high-precision clinical applications.

#### Point-of-care testing (POCT) and wearable diagnostic devices

Although high-throughput, data-driven aptamer strategies can generate larger datasets and enhance our understanding of diseases, thereby improving the accuracy of molecular diagnostics, rapid, user-friendly, and real-time POCT and wearable diagnostic devices remain critically important in specific application scenarios. In response to these needs, researchers have increasingly developed aptamer-integrated POCT and wearable sensing systems, achieving significant advances in fast, sensitive, and on-site molecular diagnostics.^36–38^ In the development of POCT and wearable diagnostic devices, two aspects are particularly important: the development of device hardware systems and the design of the sample acquisition interface. In hardware system development, ensuring that sensors can operate stably in real physical environments has become a major challenge, requiring excellent anti-interference capability. Recent studies have shown that differential circuit design enables flexible organic field-effect transistor (OFET) sensors to produce drift-free signals under conditions such as mechanical stretching, temperature variation, and bias drift. This advancement improves the engineering reliability of sensors in wearable devices and ensures more stable operation.^192^ Additionally, enabling mature sensors to achieve continuous, minimally invasive, and clinically applicable monitoring in the human body is a key aspect of device development. Researchers developed an electrochemical aptamer-based sensor patch. This patch enables safe, continuous, and accurate monitoring of drug concentrations in the interstitial fluid of healthy volunteers and establishes the pharmacokinetic relationship between interstitial and blood drug levels. This approach provides a clinically feasible tool for precision dosing and enables sensors to be truly applied at the human level.^42^

### Disease-oriented molecular diagnostic applications

**Fig. 7:**
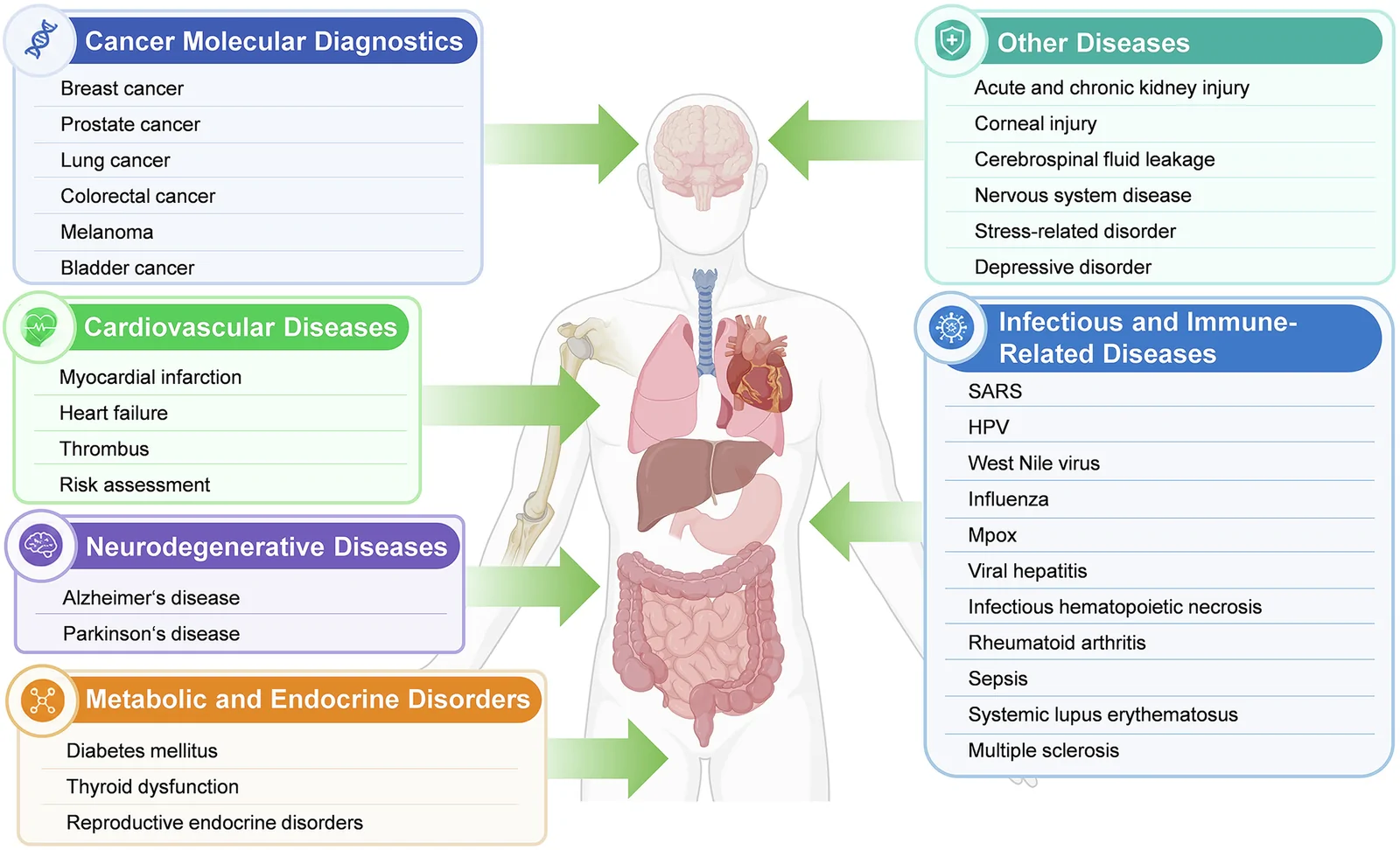
Schematic illustration of representative disease-oriented molecular diagnostic applications. This figure highlights aptamer-based diagnostics across six disease categories: (1) cancer, covering early screening, molecular subtyping, metastasis monitoring, and in vivo imaging; (2) cardiovascular diseases; (3) neurodegenerative diseases; (4) infectious and immune-related diseases, enabling pathogen identification and host immune profiling; (5) metabolic and endocrine disorders, supporting glucose and hormone monitoring for diabetes, thyroid dysfunction, and reproductive health; (6) other diseases, such as renal disorders, ophthalmological conditions, and stress-related biomarkers. Some resources in this figure come from BioRender

In terms of disease-specific challenges, as mentioned earlier, cancer remains the greatest threat to human health.^216^ Consequently, early screening, recurrence monitoring, molecular subtyping, and guidance for precision therapy continue to be major research directions in molecular diagnostics. Additionally, the sudden outbreak of severe acute respiratory syndrome (SARS) highlighted the need for enhanced emergency detection capabilities for infectious diseases, requiring the development of rapid-response platforms and the ability for on-site deployment.^217^ With the intensifying trend of population aging, the importance of chronic disease management is increasingly emphasized, necessitating long-term monitoring capabilities.^218^ In this section, we summarize recent advances in molecular diagnostic technologies across different disease contexts, aiming to provide an integrated view of the associated clinical needs and technological challenges (Fig. 7).

#### Cancer molecular diagnostics

Cancer diagnosis remains a central focus of molecular diagnostics, driving continuous innovation in tumor-targeted detection technologies.^20^ In recent years, significant advances have been achieved across multiple cancer types,^219–221^ encompassing diagnosis,^222–224^ metastasis monitoring,^225–227^ tumor typing,^84,188,228^ and therapeutic evaluation,^229,230^ with detection targets ranging from circulating biomarkers to in vivo molecular imaging.^231–237^ At the molecular level, multiplexed, label-free, and ultrasensitive detection has been achieved for emerging biomarkers such as circular RNAs (circRNAs).^238^ This platform enables the simultaneous detection of two breast cancer-related circRNAs, overcoming the limitations of traditional single-target detection. By using the unique back-splice junction (BSJ) of circRNA as the recognition target and integrating a multiplex signal amplification cascade system, the method enabled detection at ultralow concentrations. This approach enabled circRNA quantification and cell typing at the single-cell level, as well as precise diagnosis and staging of breast cancer in clinical tissue samples, demonstrating good universality and scalability. Beyond molecular detection, aptamer-based strategies have been extended to targeted tumor visualization. For instance, orally administered Raman probes (BBT-Apt@CS NPs) were engineered with AS1411 aptamers to selectively target nucleolin overexpressed on colorectal cancer cells. This strategy enables precise tumor imaging and accurate delineation of tumor margins through stacking-induced charge-transfer-enhanced Raman scattering.^239^ With the deepening of research, aptamers have been further advanced into clinical validation. Following intravesical administration, a PTK7-targeted aptamer positron emission tomography (PET) probe (⁶⁸Ga-NOTA-SGC8) was evaluated in a cohort study, in which its clinical feasibility for the specific detection of residual bladder cancer lesions was fully validated.^221^ This aptamer probe enabled highly specific molecular imaging and clearly distinguished tumor lesions from postoperative inflammation. As a result, its diagnostic accuracy was significantly superior to that of conventional fluorodeoxyglucose (FDG) PET. The advantages of this probe were particularly evident in non-muscle-invasive bladder cancer and in restaging after transurethral resection. The successful validation of this radionuclide imaging approach in a clinical cohort provides important data supporting the further clinical translation of aptamers. Collectively, these developments highlight the expanding role of aptamers from sensitive molecular detection to clinically validated imaging, underscoring their significant potential in precision oncology.

#### Cardiovascular diseases

Cardiovascular diseases remain the leading cause of morbidity and mortality worldwide.^240^ Beyond oncology, the versatile molecular recognition capability of aptamers has driven their expanding application in cardiovascular disorders,^241^ including myocardial infarction,^242^ heart failure,^243,244^ and atherosclerosis. The complex pathophysiology and dynamic biomarker fluctuations create a pressing need for sensitive, specific, and real-time molecular diagnostic strategies in this critically important field. In aptamer-based disease detection, the introduction of clustered regularly interspaced short palindromic repeats-associated protein 12a (CRISPR-Cas12a) as a signal amplification system has greatly expanded the potential application of aptamer-based diagnostics. An electrochemical aptamer sensor was developed, integrating aptamer recognition, CRISPR-Cas12a signal amplification, and a reduced graphene oxide (RGO)-MXene-Cu_2_O nanocomposite electrode. This system extends the CRISPR-Cas12a platform from nucleic acid detection to protein detection by using aptamers to convert protein signals into nucleic acid signals. The proposed method provides an integrable, low-cost, and highly sensitive strategy for low-density lipoprotein (LDL) detection, demonstrating strong potential for development into POCT devices. In addition to enabling cardiovascular disease detection, aptamers can also be applied to explore disease mechanisms. Using a bispecific aptamer, it was demonstrated for the first time that only the “activated” growth differentiation factor 11/8 (GDF11/8) isoforms are significantly associated with cardiovascular events, mortality, and dementia risk, whereas total protein levels measured by conventional mass spectrometry show no such association.^50^ Through aptamer-based detection combined with biochemical binding assays and biolayer interferometry, it was systematically shown that the aptamer specifically recognizes mature/activated GDF11/8 but not latent forms. In a large clinical cohort of 11,609 individuals, elevated levels of active GDF11/8 were associated with a 57% reduction in cardiovascular event risk and a 67% reduction in all-cause mortality. This study revealed, for the first time at the protein activation state level, the biological significance of GDF11/8, providing a mechanistically informed and highly prognostic biomarker for cardiovascular risk assessment. It also establishes a paradigm for “protein isoform detection”, highlighting the unique value of conformation-specific aptamers in precision medicine.

#### Neurodegenerative diseases

Neurodegenerative diseases have long been a focus of molecular diagnostic research due to the significant burden they impose on families and society.^245^ Aptamers have emerged as highly versatile molecular tools for investigating these diseases, offering precise and selective recognition of disease-associated protein aggregates.^246,247^ Recent studies have demonstrated the remarkable potential of aptamers for the sensitive and specific detection of amyloid fibril proteins,^246^ and key neurotransmitters such as dopamine,^248,249^ providing crucial insights into disease onset and progression. By facilitating the early detection, quantitative monitoring, and longitudinal assessment of molecular biomarkers, aptamer-based approaches hold significant promise for improving diagnostic accuracy, guiding therapeutic interventions, and enabling personalized management strategies in neurodegenerative disorders. Conventional detection of neurodegenerative diseases often requires cerebrospinal fluid (CSF) sampling to measure biomarkers. For instance, an aptamer-functionalized mesoporous gold and gold-coated magnetic nanoparticle platform using label-free surface-enhanced SERS has been developed to detect amyloid-beta 42 (Aβ42) in CSF, utilizing the endogenous Raman fingerprint of phenylalanine (Phe) as the signal.^250^ Although Aβ concentrations in CSF are higher than those in blood, making detection relatively easier, CSF collection requires lumbar puncture, which is invasive. Therefore, noninvasive sampling methods are highly desirable. To address this, a modular artificial urine biomarker probe (AUBP) was designed, integrating an aptamer targeting amyloid-beta oligomers (AβO), a DNAzyme for signal transduction, and a nanozyme (carbon quantum dots, CQDs, as a colorimetric reporter) onto SiO₂ nanoparticles.^251^ Upon intravenous injection, circulating AβO activates the probe, triggering CQD release, which is subsequently cleared via the kidneys and detected colorimetrically in urine, enabling early diagnosis of Alzheimer’s disease (AD). This strategy overcomes the limitations of traditional AUBPs that can only detect catalytically active or highly reactive biomarkers, extending applicability to any target recognizable by an aptamer. By addressing the extremely low concentration of AβO in blood and combining in vivo signal amplification with urinary enrichment, the method achieves ultrasensitive detection. This approach provides a general platform for noninvasive (urine-based) early diagnosis, with a modular design allowing flexible replacement of aptamers for different diseases. The convenience of modular construction and colorimetric readout makes it highly suitable for home-based self-testing or primary care POCT applications.

#### Infectious and immune-related diseases

Infectious diseases remain a major global health burden, and outbreaks such as SARS have underscored the urgent need for rapid-response platforms and on-site deployment capabilities.^252^ Aptamers have emerged as powerful molecular tools for pathogen detection, particularly in viral and bacterial infections.^32,34^ In viral diseases such as SARS,^33^ human papillomavirus (HPV) infection,^31^ West Nile virus,^253^ influenza,^35^ mpox,^254^ viral hepatitis,^255^ and infectious hematopoietic necrosis,^256^ aptamers enable selective recognition of pathogens, supporting sensitive detection. Beyond viral pathogens, aptamer-based sensors have also been developed for bacterial infections, including tuberculosis^257^ and toxin-producing bacteria.^258^ For example, Yan et al.^217^ combined aptamer-based reporting elements with a hinge-mediated strand displacement reaction to construct a programmable fluorescent RNA switch, named Fast Aptamer-based Reporter for Single-nucleotide-specific Genotyping through Hybridization (FARSIGHTS), capable of rapid, enzyme-free, and highly specific detection of single-nucleotide mutations. This aptamer-engineered platform accurately discriminated SARS-CoV-2 variants, including Omicron, from Alpha, Beta, and Gamma, with 100% concordance in clinical saliva samples. Similarly, the sudden outbreak of monkeypox virus (MPXV) has caused widespread concern. Li and colleagues^254^ combined recombinase-aided amplification (RAA), T4 DNA ligase-mediated probe ligation, and RNA aptamer-driven fluorescence reporting to construct a Monkeypox Fluorescent T4-Ligase Assay (MFTA) for ultrasensitive detection of MPXV DNA. By leveraging RNA Mango aptamers to amplify fluorescence upon thiazole orange 1 (TO1) binding, this strategy detected MPXV DNA with single-copy sensitivity (1 copy/μL), offering a promising approach for rapid emerging pathogen identification. In parallel with direct pathogen detection, aptamers also play an important role in monitoring host immune responses and immune-related disorders.^259,260^ Infectious and inflammatory diseases often involve dynamic fluctuations in cytokines, chemokines, and acute-phase proteins, necessitating sensitive and real-time biomarker profiling.^252,261^ Aptamer-based platforms have demonstrated robust performance in detecting inflammatory mediators such as interleukins,^262^ offering opportunities for early sepsis diagnosis and monitoring of systemic inflammatory states.^261^ Furthermore, in autoimmune diseases, including rheumatoid arthritis,^263^ systemic lupus erythematosus,^264^ and multiple sclerosis,^265^ aptamers have been developed to recognize disease-associated autoantibodies or dysregulated immune signaling molecules.

#### Metabolic and endocrine disorders

Beyond infectious and immune-related diseases, aptamers have been applied to metabolic and endocrine disorders, where fluctuating levels of metabolites and hormones pose significant diagnostic challenges.^266^ Recent studies have shown that aptamer-based platforms can provide sensitive, specific, and real-time monitoring of key biomarkers, offering valuable tools for early detection, disease management, and personalized therapy.^39,267–270^ In diabetes,^267^ aptamer-based sensors have enabled noninvasive monitoring of pancreatic β-cell mass by targeting surface proteins such as clusterin and transmembrane p24 trafficking protein 6 (TMED6). These sensors facilitate the early detection of islet damage and insulinoma and can be combined with PET/CT (computed tomography) imaging. Nucleoside modifications further enhance ribonuclease (RNase) resistance, improving their stability in clinical applications. For thyroid disorders,^268^ solid-state nanochannel platforms functionalized with aptamers have been developed to detect thyroid-stimulating hormone (TSH). Compared with conventional ELISA, these platforms achieve up to 100-fold higher sensitivity, allowing accurate quantification in serum samples from patients with thyroid carcinoma. In reproductive endocrinology,^269^ aptamer-triggered DNA hydrogel assembly within MXene-based nanofluidic membranes enables ultrasensitive detection of estradiol at femtomolar concentrations. This approach allows noninvasive hormone profiling in saliva across menstrual cycles, providing insights into fertility status and endocrine function. These advances highlight the potential of aptamer-based diagnostics to address the challenges of dynamic biomarker monitoring in metabolic and endocrine diseases.

#### Other diseases

Beyond the major disease categories outlined above, aptamer-based platforms have demonstrated broad applicability across diverse clinical contexts, underscoring their versatility in molecular diagnostics.^270–277^ In renal disorders, aptamers enable early detection of biomarkers associated with acute and chronic kidney injury, facilitating timely identification of nephrotoxicity and real-time therapeutic monitoring.^272,276^ In ophthalmology, aptamer-functionalized biosensors support rapid and sensitive detection of corneal injury markers, enabling timely intervention and improved patient outcomes.^273^ More broadly, aptamer-based assays provide quantitative evaluation of therapeutic responses across disease settings, aiding treatment optimization.^278^ In neurosurgical and neurological applications, aptamer-based approaches have been employed to detect cerebrospinal fluid leaks, monitor neurochemical changes, and track disease progression, offering minimally invasive options for longitudinal patient monitoring.^274,279,280^ In reproductive health, they enable noninvasive profiling of hormone levels and gamete quality, supporting fertility evaluation and personalized management.^270^ Moreover, aptamer platforms have been extended to aging and stress-related studies, enabling sensitive detection of biomarkers associated with oxidative stress, cellular senescence, and neuroendocrine regulation.^271,275,281^ Emerging applications in psychiatric and behavioral disorders further highlight their potential for monitoring neurotransmitters and hormone fluctuations, providing more objective tools for disease assessment and therapeutic evaluation.^275,277^ These studies collectively illustrate the adaptability of aptamers across a wide spectrum of biomedical applications, positioning them as versatile tools for sensitive, rapid, and precise molecular diagnostics beyond traditional clinical domains and offering new opportunities for precision medicine.

## New methods for molecular therapeutics

In line with their advances in molecular diagnostics, aptamers have also emerged as promising agents for molecular therapeutics.^4,5,282,283^ Compared to antibody-based therapeutics, aptamers offer significant advantages due to their storage stability in vitro and extremely low immunogenicity.^26^ Despite these favorable properties, however, the number of clinically approved aptamer drugs remains extremely limited, highlighting key translational challenges.^23,284^ In this section, we summarize representative aptamer therapeutics and emerging strategies aimed at addressing these challenges, providing insights to advance the clinical development of aptamer-based therapies.

### Mechanism of aptamers in molecular therapeutics

Several distinct design strategies have been developed for constructing aptamer-enabled therapeutic systems.^23^ Aptamers can function directly as therapeutic agents by disrupting receptor‒ligand interactions or modulating disease-associated signaling pathways.^35,53,285^ Alternatively, they can be conjugated with small-molecule drugs to form aptamer-drug conjugates (ApDCs), serving as antibody substitutes for targeted therapy.^26^ Beyond small-molecule conjugation, aptamers have been incorporated into larger functional platforms.^286,287^ Coupling with bioactive peptides or DNA nanostructures provides macromolecular systems with selective targeting capability, while integration with a wide range of nanomaterials has become one of the most extensively explored directions in the field.^288,289^ In these nanocarrier-based systems, aptamers function as targeting ligands that enhance delivery efficiency and improve therapeutic selectivity. Furthermore, leveraging their intrinsic programmability, aptamers and nucleic acid architectures can be engineered into self-assembled systems capable of executing advanced biological functions, such as targeted protein degradation and immune modulation.^290–292^

### Aptamer-based therapy strategies

**Fig. 8:**
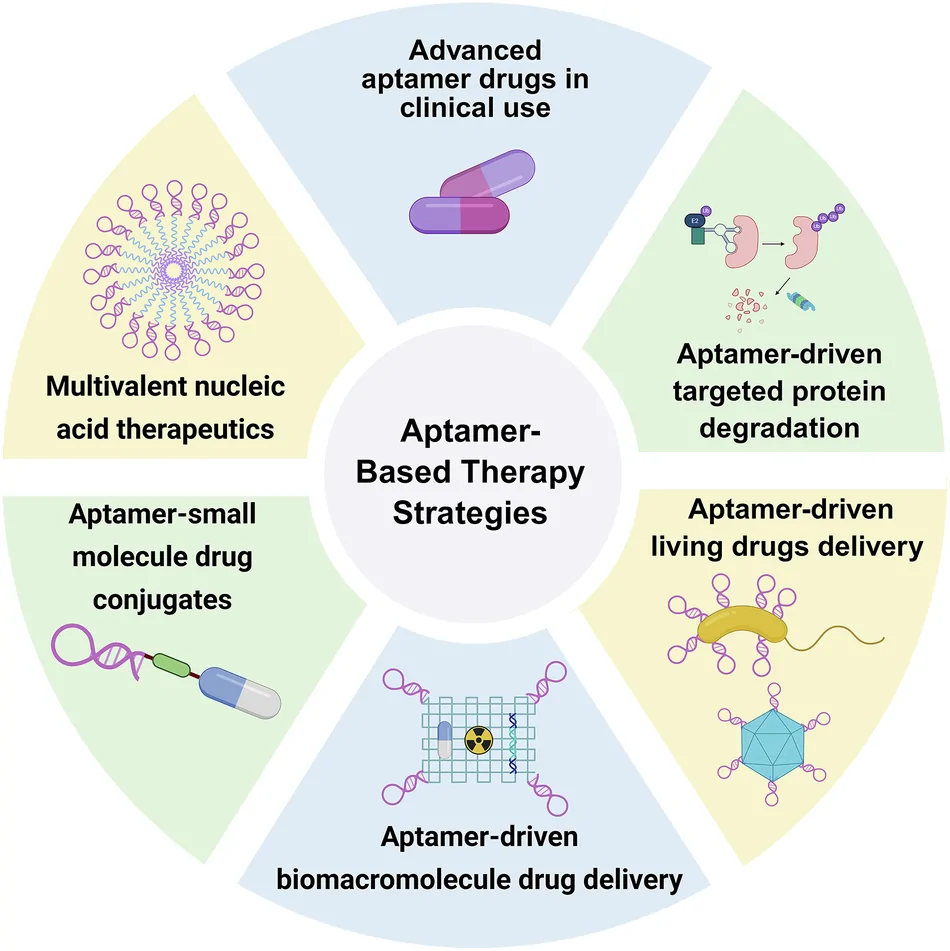
Schematic overview of aptamer-based therapeutic strategies. This figure summarizes six major aptamer-based therapeutic approaches: (1) multivalent nucleic acid therapeutics using DNA nanostructures or scaffolds for enhanced avidity and synergistic delivery; (2) aptamer-small-molecule drug conjugates for targeted chemotherapy, photodynamic therapy, and combination regimens; (3) aptamer-driven biomacromolecule delivery for precise protein/nucleic acid transport; (4) aptamer-driven living drug delivery using engineered bacteria or viruses for tumor penetration and immune activation; (5) aptamer-driven targeted protein degradation via bispecific chimeras recruiting proteasomal or lysosomal pathways; (6) advanced aptamer drugs in clinical use, including FDA-approved pegaptanib and avacincaptad pegol, alongside ongoing clinical trials of novel ApDCs. Some resources in this figure come from BioRender

To overcome current limitations and fully harness the therapeutic potential of aptamers, extensive efforts have focused on developing advanced design strategies.^23,26^ Multivalent aptamer constructs and macromolecular conjugation have been explored to enhance binding stability, optimize intracellular trafficking, and broaden therapeutic scope.^286,293^ Efforts to link aptamers with small-molecule drugs have enabled the replacement of antibodies and facilitated the efficient development of novel aptamer-drug conjugates.^26^ Meanwhile, integration with biological carriers such as bacteria or viruses leverages their intrinsic delivery and metabolic properties, endowing aptamer-based systems with additional functionalities.^294,295^ Furthermore, by combining aptamers with the programmability of nucleic acids, engineered DNA architectures have enabled sophisticated functions, including targeted protein degradation.^296–298^ This section reviews recent representative studies in these areas, providing a reference for the development of next-generation aptamer-based therapeutics **(**Fig. 8**)**. Table 5 summarizes the advanced aptamer-based therapeutic strategies.

**Table 5:** Summary of advanced aptamer-based therapeutic strategies

Strategy category	Key technologies	Representative advances	Key advantages	Quantitative performance	Clinical translation stage
**Multivalent nucleic acid therapeutics**	Programmable DNA nanostructures; Self-assembled DNA nanoclusters; Nucleic acid scaffolds; Nanomaterial conjugation; Natural materials	Cyclic single-stranded DNA scaffold integrating aptamers with small-molecule drug for immune cell recruitment, tumor killing, and innate immune activation^293^; Y-shaped DNA scaffold-based bispecific aptamer targeting distinct epitopes of SARS-CoV-2 RBD for synergistic neutralization^310^; Upconversion nanoparticles loaded with DOX and conjugated with TfR1-targeting multivalent aptamers; NIR-induced covalent anchoring to tumor surface prolongs retention and enhances immunogenic cell death^307^; Natural pollen microcapsules encapsulating dual aptamers via disulfide bonds for ROS-responsive release in rheumatoid arthritis dual-targeted therapy^305^	Enhanced binding avidity; expanded target coverage; improved tumor retention; codelivery of multiple drugs; synergistic activation of innate and adaptive immunity	Tumor growth inhibition >90%^293^; SARS-CoV-2 pseudovirus neutralization IC50 8.2 nM^310^; Tumor DOX accumulation: >6-fold increase at 6 h, >5.5-fold at 120 h vs. noncovalent nanodrugs; Tumor inhibition rate: ~70%^307^; tk-Apt@pollen: TNFα level reduced from ~69 pg/mL to ~11 pg/mL in mouse serum^305^	Preclinical (in vivo efficacy in multiple tumor models)
**Aptamer-small-molecule drug conjugates**	Covalent conjugation; Intercalation into duplex regions; Carrier-free DNA nanostructure coassembly; Liposome encapsulation	PTK7-targeting ApDC (Sgc8c-M) with MMAE payload; superior efficacy to paclitaxel and matched ADCs in CDX/PDX models; favorable PK/TK and safety in cynomolgus monkeys^320^; Photosensitizer PA precisely conjugated to PTK7 aptamer Sgc8c via solid-phase synthesis and copper-free click chemistry; tunable photophysical activity^312^; Camptothecin and photosensitizer HPPH conjugated to aptamer and antisense oligonucleotide, respectively; coassembled into carrier-free nanoparticles for trimodal chemo-photo-gene therapy^308^; Lipo-Apt system: drug-binding aptamers inside liposomes provide an additional affinity barrier, enabling affinity- and diffusion-controlled dual release; prolonged drug action and reduced systemic toxicity^313^	Simple synthesis; low immunogenicity; tunable drug loading; wide therapeutic window; combination therapy (chemo-photo-gene); sustained and controlled release	Exploration on cynomolgus monkeys^320^; Aptamer-based photothermal therapy for enhanced treatment efficiency^312^; Apt-CHA (three-in-one): Tumor inhibition rate ~80%^308^; Lipo-Apt-TTX: Sensory nerve block duration 157.4 h; 4.1-fold longer than Lipo-TTX alone^313^	Sgc8c-M: Phase 1 clinical trial (NCT06687941, GPC3-targeting ApDC AST-201)^320^; Sgc8c-1PA, Apt-CHA, Lipo-Apt-TTX: Preclinical
**Aptamer-driven biomacromolecule drug delivery**	Aptamer-peptide conjugation; Aptamer-oligonucleotide conjugation; DNA nanostructure scaffolds	DNA nanostructures simultaneously loading doxorubicin and ribonucleoproteins for multifunctional aptamer-based molecular therapy^286^	Strong recognition capability; functional and structural versatility; codelivery; spatiotemporal controlled release	Doxorubicin loading capacity: high (DNA tetrahedron framework); Cell viability reduction: ~71.7%^286^	Preclinical
**Aptamer-driven living drug delivery**	Engineered EVs; Click chemistry-based bacterial surface anchoring; Oncolytic virus delivery	PSMA-targeted engineered EVs modified with ROS-responsive PD-L1-blocking aptamers on the surface and loaded with oncolytic virus inside; intravenous administration enables targeted delivery and microenvironment-responsive release, overcoming the need for intratumoral injection and PD-L1 upregulation-induced resistance^294^; Click chemistry covalent anchoring of PTK7-targeting ApDC (Sgc8c-MMAE) onto attenuated Salmonella typhimurium VNP20009 surface; living bacteria-drug complex (VNP@Sgc8c-MMAE) combines bacterial hypoxia tropism with aptamer active targeting, achieving triple synergy: deep penetration, enhanced ApDC stability, and bacterium-induced immune activation^295^	Intravenous administration feasible; localized immune modulation; deep tumor penetration; enhanced ApDC stability; bacterium-induced immune activation	OH2@RRA-AP-EVs: Tumor growth inhibition: ~70% vs. free OH2; CD8 + T-cell infiltration: >2-fold increase vs. control^294^; ApDCs surface density: ~2.5 nmol/10⁸ CFU (~1.5 × 10⁷ molecules/bacterium); Serum stability: >60% intact at 48 h; Tumor colonization: ~90% at 72 h; TGI: ~70–80% (subcutaneous, PDX, in situ models); CD8⁺ T-cell increase: ~3-fold^295^	Preclinical
**Aptamer-driven targeted protein degradation**	PROTAC (recruiting E3 ubiquitin ligases); LYTAC (lysosome targeting); Light-induced autophagic degradation	Photoactivatable bispecific aptamer chimera targeting PTK7 and IGFIIR with a near-infrared photosensitizer; induces ROS-triggered autophagy to enhance protein clearance^298^	Complete protein elimination; overcomes drug resistance; addresses undruggable targets; leverages cellular endogenous disposal machinery	Membrane protein degradation efficiency: enhanced via phototriggered autophagy^298^	Preclinical
**Advanced aptamer drugs in clinical use**	FDA-approved aptamer drugs	Pegaptanib (Macugen^®^, approved 2004): RNA aptamer targeting VEGF for neovascular age-related macular degeneration^53^; Avacincaptad pegol (Izervay^®^, approved 2023): aptamer targeting the complement system for geographic atrophy^54^	First FDA-approved aptamer (2004); 2023 approval marks a critical revival; advances in chemical modifications and delivery strategies are driving a new wave of development	Pegaptanib: K_*d*_ ~ 0.5 nM (VEGF); Avacincaptad pegol:*K*_d_ ~ 0.1 nM	FDA-approved (2004, 2023); >71 aptamer-related reagents in clinical trials (as of March 15, 2026)

#### Multivalent nucleic acid therapeutics

Multivalent nucleic acid therapeutics have emerged as a promising strategy to overcome the intrinsic limitations of monovalent aptamer designs in cancer therapy.^299,300^ Tumor heterogeneity, reflected by diverse cellular subpopulations and dynamic antigen expression, often limits the efficacy of single-target aptamers.^301^ Rapid systemic clearance, poor retention at tumor sites, and the reversible nature of monovalent aptamer–target interactions further constrain their therapeutic potential.^301^ Multivalent designs address these challenges by integrating multiple aptamer units within a single construct, thereby enhancing binding avidity, expanding target coverage, and improving tumor retention.^302,303^ These multivalent constructs can be engineered using programmable DNA nanostructures,^286^ self-assembled DNA nanoclusters,^293^ or nucleic acid scaffolds,^304^ which allow precise spatial arrangement and controlled valency. Such structural tunability enables simultaneous recognition of multiple tumor-associated biomarkers,^302^ improving selectivity within heterogeneous tumor environments. In parallel, conjugation with nanomaterials further increases local aptamer density and promotes cooperative binding.^305,306^ Beyond improved targeting, multivalent systems can facilitate payload delivery or directly modulate biological processes.^293,307^ As a result, they function as versatile platforms capable of acting as standalone nucleic acid therapeutics, inducing receptor clustering and signaling interference, or acting as carriers for chemotherapeutic and immunomodulatory agents.^308,309^

Building on these design principles, several studies have illustrated how multivalent nucleic acid platforms can enhance binding avidity, tumor retention, and functional versatility, thereby improving therapeutic outcomes in preclinical cancer models.^26^ DNA nanostructures, with their inherent programmability, have been widely employed to construct multivalent aptamer therapeutics. For example, DNA-templated polymerization enables cyclic single-stranded multivalent aptamer-drug conjugates that integrate multiple aptamers (e.g., programmed cell death protein 1 (PD-1), PD-L1, and c-Met) with three drugs (monomethyl auristatin E (MMAE), the Toll-like receptor 7/8 (TLR7/8) agonist T785, and the stimulator of interferon genes (STING) agonist diABZI) within a single cyclic DNA scaffold.^293^ This design enables triple functionality: immune cell recruitment, tumor cell killing, and innate immune activation. It addresses key challenges in cancer immunotherapy, including insufficient T-cell infiltration, immunosuppressive tumor microenvironments, and tumor heterogeneity. By simultaneously targeting T cells (PD-1) and tumor cells (PD-L1/c-Met) with multiple specific aptamers and releasing both chemotherapeutic agents and innate immune agonists. This strategy synergistically activates both innate and adaptive immunity. Two aptamers targeting distinct epitopes of the SARS-CoV-2 receptor-binding domain (RBD) (CoV-2-1C and CoV-2-6C3) were anchored onto a Y-shaped DNA scaffold to form a bispecific aptamer, Aptx2-L, a long-arm Y-shaped bispecific aptamer construct, enabling synergistic multiepitope binding to the viral spike protein.^310^ This design mitigates the risk of viral escape caused by mutations that compromise single-epitope neutralization, while the spatially matched Y-shaped scaffold mimics the trimeric structure of the virus, promoting cooperative dual-epitope binding, enhancing binding affinity, and reducing the likelihood of mutation-mediated evasion. Additionally, to enhance the binding of aptamers to target molecules, a diazirine-modified multivalent aptamer targeting transferrin receptor 1 (TfR1) was conjugated onto the surface of upconversion nanoparticles (UCNPs) loaded with doxorubicin (DOX).^307^ Upon near-infrared (NIR) light irradiation, the aptamer forms covalent crosslinks with TfR1 on the tumor cell surface, achieving prolonged intratumoral retention. In contrast, conventional nanomedicines exhibit short tumor retention times and rely solely on the enhanced permeability and retention (EPR) effect, which limits delivery efficiency. By using light-induced covalent bond formation to “anchor” the nanomedicine on tumor cell surfaces, the retention time is significantly extended. Enhanced DOX delivery induces stronger immunogenic cell death (ICD), thereby activating antitumor immune responses. In addition to conventional synthetic materials for constructing multivalent nucleic acid therapeutics, natural materials, owing to their intrinsic advantages, also provide valuable inspiration for developing multivalent nucleic acid therapeutic strategies. Natural pollen microcapsules have been used as carriers to encapsulate two aptamers and are endowed with reactive oxygen species (ROS)-responsive release via disulfide bonds, enabling dual-targeted therapy for rheumatoid arthritis (RA).^305^ This approach effectively addresses several challenges, including the complex pathology of RA, the limited efficacy of single-target therapies, the susceptibility of aptamers to nuclease degradation and rapid clearance in vivo, and the lack of efficient and stable delivery systems. By leveraging the inherent protective structure of pollen and ROS-responsive release, aptamers can be precisely delivered to the inflammatory microenvironment.

#### Aptamer-small molecular drug conjugates

Aptamers have antibody-like tumor-recognition capability but offer easier synthesis and modification with lower immunogenicity, making them promising alternatives for ApDCs in cancer therapy.^311^ Aptamers can be linked to small-molecule drugs via covalent conjugation or intercalation into duplex regions, enabling chemotherapy and photodynamic therapy.^108,312–315^ Beyond the conventional use of aptamer-small-molecule chemotherapeutics, aptamer-conjugated photodynamic agents provide important insights for aptamer-based therapeutic strategies. Photoactive ApDCs are becoming a promising approach in the context of ApDCs. To date, Ce6,^316–318^ pyrochlorophyll A (PA),^173^ and Ru^298,312,319^ have been covalently modified on aptamers for precision cancer therapy. By combining solid-phase DNA synthesis, PA was precisely conjugated to the PTK7 aptamer Sgc8c via solid-phase synthesis and copper-free click chemistry, improving solubility and photodynamic activity. The study showed that ApDC photophysical properties can be tuned by controlling drug loading without compromising targeting ability.^312^ With the advancement of ApDCs, comprehensive preclinical evaluation has become essential for clinical translation. A PTK7-targeting ApDC (Sgc8c-M) was systematically evaluated for pharmacokinetics, toxicokinetics, safety, and antitumor efficacy from rodents to cynomolgus monkeys.^320^ Using MMAE as the payload, Sgc8c-M showed enhanced antitumor activity across multiple cell line-derived xenograft (CDX) and patient-derived xenograft (PDX) models and outperformed paclitaxel and target-matched ADCs. Favorable pharmacokinetic/toxicokinetic (PK/TK) profiles, sustained tumor retention, a wide therapeutic window, and good safety support its clinical translation.

In recent studies, ApDCs have been further advanced by combining them with nanomaterials^307,308,313^ or by constructing DNA nanostructure-based ApDC assemblies,^293^ effectively improving drug delivery performance. The self-assembly of DNA nanostructures and their facile conjugation with aptamers enable the construction of carrier-free ApDCs for drug delivery. To avoid the complexity and toxicity of conventional multidrug carriers, a carrier-free nanoparticle platform was developed through the coassembly of chemically modified drug-DNA conjugates, enabling combination therapy.^308^ In this system, light-triggered ROS generation promotes lysosomal escape, followed by glutathione-responsive intracellular drug release. Chemotherapeutic camptothecin and the photosensitizer 2-[1-hexyloxyethyl]-2-devinyl pyropheophorbide-a (HPPH) were conjugated to a targeting aptamer and an antisense oligonucleotide, respectively, and coassembled into a carrier-free DNA nanostructure, enabling trimodal chemo-, photo-, and gene therapy. Combining aptamers with nanomaterials, especially liposome-based ApDCs, is a common strategy. In Lipo-Apt systems, drug-binding aptamers inside liposomes provide an additional affinity barrier, delaying drug release beyond the liposome’s physical barrier.^313^ This approach overcomes conventional liposome limitations, such as burst release and short efficacy, by combining affinity and diffusion-controlled release, prolonging drug action and reducing systemic toxicity.

#### Aptamer-driven biomacromolecule drug delivery

In drug delivery, aptamers have demonstrated remarkable versatility beyond their conventional role in ApDCs.^45,287,321^ Recent advances increasingly focus on conjugating aptamers with biomacromolecules, including peptides^289^ (as targeting motifs), oligonucleotides^321,322^ (for gene modulation or immunoactivation) and DNA nanostructures^286–288^ (as programmable scaffolds for multivalent binding and controlled release). Such integrations can combine the molecular recognition capability of aptamers with the functional and structural versatility of macromolecular cargos. They are precise, target-specific delivery and more sophisticated therapeutic strategies, achieving codelivery of multiple agents and spatiotemporally controlled release within the tumor microenvironment.^286,287,321^ Owing to the designability and ease of assembly of DNA nanostructures, they have become commonly used biomacromolecules for aptamer-driven drug delivery. For example, chemotherapeutic agents such as doxorubicin can be loaded into the double strands of DNA nanostructures and, when codelivered with ribonucleoproteins, enable facile multifunctional aptamer-based molecular therapies.^286^ Importantly, these hybrid systems can overcome key limitations of small-molecule conjugates, such as rapid renal clearance, short circulation time, and insufficient tumor retention.^45,322^ However, the increased structural complexity and nanoscale size of these constructs introduce challenges inherent to nanomedicine, including manufacturing reproducibility, in vivo uncertainty, and barriers to clinical translation.

#### Aptamer-driven living drug delivery

Engineered bacteria and viruses, as living therapeutics, possess intrinsic biological features, enabling them to navigate physiological barriers and actively infiltrate the immunosuppressive tumor microenvironment.^323–325^ Beyond their role as delivery vehicles, these living agents can elicit antitumor immune responses, effectively converting “cold” tumors into immunologically active “hot” tumors and enhancing therapeutic efficacy.^295,326^ In studies involving virus-based therapy, PSMA-targeted engineered EVs were constructed, with ROS-responsive PD-L1-blocking aptamers modified on the surface and oncolytic viruses loaded inside, enabling tumor-targeted delivery after intravenous administration and microenvironment-responsive immune checkpoint blockade.^294^ This strategy addresses several limitations of conventional oncolytic virotherapy, including the requirement for intratumoral injection, which limits treatment of metastatic lesions, and the upregulation of PD-L1 induced by oncolytic virus therapy, which leads to adaptive immune resistance. Although combination therapy with PD-L1 antibodies can be effective, it is often associated with high systemic toxicity. By using EVs for targeted delivery of the oncolytic virus and ROS-responsive local release of PD-L1 aptamers, this approach enables intravenous administration and localized immune modulation, thereby improving the therapeutic efficacy and safety of oncolytic virotherapy. Although the aptamer-enabled targeted delivery of the oncolytic virus, it was not directly conjugated to the virus, which inevitably increased the complexity of the delivery system design and assembly. One possible reason is the relatively small size of the virus, which makes direct conjugation challenging. In contrast, bacteria have a relatively larger size, making direct conjugation with aptamers more feasible. Through click chemistry, a PTK7-targeting aptamer-drug conjugate (Sgc8c-MMAE) was covalently anchored onto the surface of attenuated Salmonella typhimurium VNP20009, constructing a living bacteria-drug complex (VNP@Sgc8c-MMAE).^295^ This system combines the tumor hypoxia tropism of bacteria with the active targeting capability of aptamers to achieve synergistic therapy. This strategy addresses several challenges in pancreatic cancer treatment. The dense stroma limits drug penetration and reduces the effectiveness of conventional delivery. In addition, ApDCs are vulnerable to nuclease degradation and rapid in vivo clearance, compromising their stability, while bacterial therapy alone shows limited targeting and insufficient therapeutic efficacy. By using bacteria to carry ApDCs, this platform achieves three synergistic effects: deep tumor penetration, enhanced ApDC stability, and bacterium-induced immune activation.

#### Aptamer-driven targeted protein degradation

The significance of targeted protein degradation lies in its ability to eliminate pathogenic proteins entirely rather than merely inhibiting their function. It offers a powerful strategy to address “undruggable” targets and overcome drug resistance by leveraging the cell’s own disposal machinery.^297,327^ Due to their high affinity and specificity for protein targets, aptamers have recently attracted considerable attention as recognition modules for targeted protein degradation strategies.^291,298,328,329^ Increasing efforts have focused on harnessing aptamers to bind specific proteins and exploiting the mechanisms of proteolysis-targeting chimeras (PROTACs)^109,330^ and lysosome-targeting chimeras (LYTACs)^331–333^ to achieve selective protein degradation. In PROTAC-based approaches, beyond relying on conventional small-molecule ligands to recruit E3 ubiquitin ligases,^334,335^ researchers have further identified and developed aptamers capable of binding ubiquitination-related proteins.^296,336^ In LYTAC-based strategies, efficient lysosomal trafficking of target proteins is essential for degradation. To promote internalization and lysosome delivery, multiple approaches have been explored, including aptamer-mediated endocytosis,^337^ glycan-mediated endocytosis,^338^ size-dependent endocytosis,^292,339^ DNA nanostructure-assisted internalization,^290,340^ and nanoparticle-facilitated uptake^333,341^. These strategies have collectively demonstrated promising results in enhancing lysosomal targeting and degradation efficiency. Beyond the examples mentioned, the concept of using external physical stimuli to enhance protein degradation has also been explored.^298^ A photoactivatable bispecific aptamer chimera targeting PTK7 and insulin-like growth factor II receptor (IGFIIR) with a near-infrared photosensitizer induced ROS-triggered autophagy, offering a novel strategy to enhance protein clearance.

#### Advanced aptamer drugs in clinical use

With continued advances, aptamer-based molecular therapeutics have demonstrated unique advantages across multiple areas, laying an important foundation for clinical translation. This was exemplified by AS1411, a nucleolin-targeting aptamer. Although initially promising, it ultimately failed to demonstrate sufficient efficacy in phase II studies and was discontinued, highlighting the substantial hurdles in aptamer drug development.^342^ The first aptamer to achieve FDA approval was pegaptanib (Macugen) in 2004, an RNA aptamer targeting VEGF for neovascular age-related macular degeneration.^53^ While this milestone validated the therapeutic potential of aptamers, pegaptanib struggled commercially against more effective antibody-based therapies, leaving the field in a prolonged lull. In 2023, avacincaptad pegol (Izervay) was approved for geographic atrophy, targeting the complement system, marking a critical revival for aptamer therapeutics.^54,285^ According to clinical trial data (ClinicalTrials.gov), as of March 15th, 2026, more than 71 aptamer-related reagents had entered clinical testing. This long-awaited approval underscores renewed optimism for the modality. With advances in chemical modifications, delivery strategies, and regulatory pathways, the field is now poised for a new wave of aptamer therapeutics, offering hope that more candidates will successfully navigate the clinic and reach patients.

Currently, no ApDCs have been approved for clinical cancer therapy. Encouragingly, several aptamers targeting different cancer markers are currently under clinical investigation. In 2025, AST-201, an ApDC, was utilized in a phase 1 clinical study involving patients with GPC3-positive advanced solid tumors. This study aims to evaluate the safety, tolerability, and preliminary efficacy of AST-201, targeting GPC3-positive advanced solid tumors (NCT06687941). Furthermore, recent research has increasingly focused on optimizing the structure of ApDCs to enhance their in vivo stability and molecular targeting, addressing the needs for clinical translation.^295,320,343,344^ These application-oriented studies have significantly advanced the clinical development of ApDCs.

### Disease-oriented molecular therapeutic applications

Compared to aptamer-based molecular diagnostics, the development of molecular therapeutics has lagged slightly behind, yet it has also been investigated just as extensively.^4,5^ Consequently, similar to molecular diagnostics, molecular therapeutics are predominantly focused on cancer treatment, with research on cancer vastly outnumbered by that on other diseases. In the context of infectious disease treatment, aptamers offer distinct advantages for viral therapy, being superior to antibodies in both size and immunogenicity.^106,345^ Regarding immune disorders, cardiovascular diseases, neurodegenerative diseases, metabolic disorders, and other conditions, although numerous disease-specific aptamers have indeed been developed for diagnostic purposes, the substantial differences between therapy and detection mean that diagnostic aptamers often cannot be directly repurposed for treatment, necessitating rescreening and new development.^51,346–348^ This section reviews representative research findings in these areas, aiming to provide a reference for the future design and development of aptamer-based molecular therapeutics.

#### Cancer molecular therapeutics

Cancer remains the central focus of molecular therapeutics.^216,349,350^ Aptamer-based strategies have attracted increasing attention with the emergence of diverse approaches across multiple tumor types,^23,26^ including melanoma,^351–353^ breast cancer,^354–356^ colorectal cancer,^357^ pancreatic cancer^358,359^ and so on.^360,361^ Notably, the field has shifted from conventional cytotoxic drug delivery toward immune-oriented strategies,^362,363^ such as tumor vaccines^356^ and immune activation,^352,355^ in line with broader trends in oncology. Aptamer-enabled tumor vaccines targeting cancer stem cells (CSCs), key drivers of recurrence and metastasis, represent a particularly promising direction. Based on aptamer technology, a nanovaccine was developed using nanovesicles derived from aldehyde dehydrogenase 1 family member A1 (ALDH1A1)-overexpressing tumor cell-derived nanovesicles (ANVs), which were loaded with YTHDF1-targeting small interfering RNA (siYTHDF1) as an epigenetic nanoregulator and modified with a dendritic cell-specific intercellular adhesion molecule-3-grabbing nonintegrin (DC-SIGN)-targeting aptamer.^363^ This vaccine suppresses YTH N6-methyladenosine RNA-binding protein 1 (YTHDF1) expression in dendritic cells, thereby reducing lysosomal protease-mediated degradation of internalized antigens and enhancing antigen cross-presentation. As a result, it activates immune responses against both cancer stem cells and bulk tumor cells and induces durable immune memory.

In addition, aptamer-based strategies for promoting immune activation have shown considerable potential in the field of molecular therapeutics. Particularly when combined with DNA nanostructures, they offer greater flexibility in designing molecular therapeutic tools. For example, insufficient T-cell infiltration in solid tumors remains a major barrier to immunotherapies, including chimeric antigen receptor T-cell (CAR-T) and immune checkpoint inhibitors. Monocytes are naturally recruited to tumors, but conventional methods struggle to stably link T cells with monocytes. Aptamer-modified DNA tetrahedra were employed as intercellular nanoconnectors to specifically link cluster of differentiation 8-positive (CD8⁺) T cells with lymphocyte antigen 6 complex locus C-positive (Ly6c⁺) monocytes, forming T-cell-monocyte assemblies.^362^ By leveraging the natural tumor-homing ability of monocytes, T cells were effectively hitchhiked into the tumor core, resulting in significantly enhanced T-cell infiltration. Aptamer-mediated radionuclide therapy offers high tumor specificity, rapid tissue penetration, and low immunogenicity. Its favorable pharmacokinetic properties enable precise and effective targeted radiotherapy while minimizing off-target toxicity. These advantages make it a promising avenue in aptamer-based molecular therapeutics. Recently, a PTK7-targeting aptamer was integrated with a DNA tetrahedral framework and subsequently complexed with the radionuclide ^177^Lu, establishing an aptamer-guided radionuclide therapeutic strategy.^172^ Collectively, these advances highlight the evolving paradigm of aptamer-based cancer therapeutics, from passive targeting to active immune modulation and multifunctional precision therapy.

#### Cardiovascular diseases

Although cardiovascular disease is the leading cause of death worldwide, aptamer-based molecular therapeutic strategies for cardiovascular diseases are rarely reported.^348,364^ This may be attributed to the current lack of suitable targeting aptamers for cardiovascular conditions. Cardiovascular diseases often occur in areas with high and rapid blood flow, where the recognition and binding of ligands with targeting capabilities are highly susceptible to interference from fluid shear stress. Consequently, it is challenging to screen aptamers appropriate for cardiovascular disease therapy. A recently reported smart DNA nanoflower (sDNF) platform was constructed using aptamers and functionalized with neuron-targeting aptamers.^348^ This design enabled specific targeting of sympathetic ganglia, reducing ventricular arrhythmias and alleviating neuropathic pain following myocardial infarction. However, this approach does not provide effective therapeutic intervention for the disease itself.

#### Neurodegenerative diseases

Currently, the understanding of neurodegenerative diseases such as Alzheimer’s disease and Parkinson’s syndrome remains insufficient, and there is an extreme lack of methods to treat or even delay their progression.^365^ Although some drugs have been approved for these conditions, treatment strategies are still in the preliminary exploratory stage.^365^ Similarly, while aptamer-based detection technologies for neurodegenerative diseases are emerging rapidly, therapeutic strategies remain scarce. The affected regions in neurodegenerative diseases are located in the brain, and the development of aptamers capable of entering the brain is a prerequisite for nucleic acid drugs to achieve brain delivery.^366,367^ Two recent studies based on aptamers for Alzheimer’s disease treatment provide references for further attempts to develop aptamer-based molecular therapeutics for neurodegenerative diseases. Zhuo et al.^51,52^ developed a method to clear extracellular Tau protein in Alzheimer’s disease based on a Tau-targeting aptamer. Tau-specific aptamer-conjugated monocytes cross the blood‒brain barrier and enter Tau-rich brain regions. They clear extracellular Tau, reduce neuroinflammation, protect neurons, and improve memory and cognition, demonstrating direct therapeutic targeting of Tau in neurodegenerative disease. Another study used multiomics approaches to identify a serum protein signature linked to neuroprotection.^368^ This research modulates signaling pathways involved in lipid metabolism and inflammation, offering a potential strategy to treat neurodegenerative diseases through pathway intervention.

#### Infectious and immune-related diseases

Leveraging the high specificity of aptamers enables efficient identification of infectious diseases, offering significant advantages over antibiotic-resistant infections and neutralizing antibodies that may induce immune responses.^23^ This approach can achieve pathogen killing by interfering with microbial metabolism or by delivering small-molecule drugs in a targeted manner.^369,370^ Recently, aptamers have demonstrated promising efficacy in anti-infective research. In antiviral studies, they not only inhibit a broad spectrum of viral infections but also show effectiveness against specific viruses, such as rabies and influenza pneumonia.^106,345,371^ In antibacterial research, aptamers have also yielded considerable therapeutic outcomes.^372,373^ However, it is worth noting that the development of small-molecule drugs for viral infections currently lags far behind the clinical need for treatment, making aptamers a promising alternative strategy. In contrast, a wide variety of antibacterial agents are already available, and except for certain drug-resistant bacteria, most infections can be effectively managed. Therefore, the development of aptamer-based antibacterial therapies presents both opportunities and challenges.

Recent advances in aptamer-based therapies for immune-related diseases have focused on two main areas. One area involves the treatment of organ-specific immune disorders, including dry eye disease,^374^ psoriasis,^375^ inflammatory bowel disease,^376^ and Graves’ ophthalmopathy^175^. The other area focuses on arthritis and rheumatoid arthritis.^347,377,378^ Under these conditions, aptamer-based molecular therapies have shown promising results. Designing aptamers based on disease-specific pathological mechanisms further enhances targeting precision and speeds the development of new therapeutics. For example, Huang et al.^347^ elucidated the key role of endothelial cell-specific molecule 1 (ESM1) in promoting the invasive transformation of fibroblast-like synoviocytes in rheumatoid arthritis via ESM1-mediated pathways. Based on this mechanism, they employed SELEX technology to screen and obtain the ESM1-neutralizing aptamer ESMA04. In a collagen-induced arthritis mouse model, ESMA04, either alone or in combination with etanercept, effectively alleviated arthritic symptoms without causing hepatotoxicity or nephrotoxicity. This study provides a novel therapeutic strategy for rheumatoid arthritis using aptamers. By thoroughly investigating key proteins in the pathogenic pathway, we offer an important reference for the development of aptamers in other diseases.

#### Metabolic and Endocrine Disorders

Although the incidence of metabolic and endocrine disorders is comparably high relative to that of cancer, the development of aptamers for the treatment of these diseases remains limited. Consequently, current research on aptamer-based molecular therapeutic strategies for this category of diseases is extremely scarce compared to oncology. Recent representative studies have primarily focused on diabetes and obesity resulting from glucose and lipid metabolism disorders.^346,379–381^ For example, an aptamer-functionalized hydrogel efficiently captures and releases endogenous chemokines from the wound microenvironment, promoting macrophage recruitment to the diabetic wound site.^379^ By enriching these signals, the hydrogel expands the local macrophage pool, reactivates efferocytosis, and initiates the resolution of chronic inflammation and tissue repair. Additionally, some research has been conducted on the prostaglandin signaling pathway, a key axis in lipid mediator and endocrine signal regulation.^382^ Overall, however, studies targeting metabolic and endocrine disorders are still relatively few.

#### Other diseases

In addition to the diseases mentioned above, aptamer-mediated molecular therapies have also shown promise in other conditions. For instance, in bone defect repair, nucleic acid aptamers combined with DNA nanostructures can promote osteogenesis and vascularized differentiation through the recruitment of endogenous active molecules or the release of exogenous active factors.^383,384^ Alternatively, when integrated with exosomes, they facilitate osteogenic differentiation by scavenging reactive oxygen species, protecting mitochondrial function, and regulating macrophage polarization.^385^ In osteoporosis, aptamers have been incorporated into mineralized DNA origami patches to target inhibitory factors in bone tissue. This approach promotes osteogenesis and improves the bone microenvironment by enhancing reactive oxygen species clearance.^386^ Furthermore, nucleic acid aptamers have also been explored in therapeutic research for conditions such as otitis media, liver fibrosis, and muscular atrophy/dystrophy.^387–389^

## Challenges and future perspectives of nucleic acid aptamers

### Challenges and future perspectives in molecular diagnosis

Although aptamers have been extensively studied in molecular diagnostics and have achieved fruitful results, certain challenges remain before their widespread application, which are mainly manifested in the following aspects. (1) In terms of in vitro detection, target analytes are often present at low abundance. Therefore, amplifying the binding signal between the aptamer and the target remains a perpetual topic in aptamer-based detection.^20^ This necessitates the continuous development of novel signal reporter molecules and detection instruments to constantly improve signal intensity and the signal-to-noise ratio. (2) Meanwhile, although monovalent aptamers possess good recognition capability for targets, they are susceptible to interference from fluid flow, which can affect binding stability post-recognition and cause the monovalent aptamer to detach easily from the target, thereby compromising detection performance.^184,301^ This issue can potentially be addressed by developing multivalent aptamer detection or utilizing covalent proximity ligation to enhance post-recognition stability.^301^ (3) For reusable detection scenarios, such as wearable devices, the aptamer recognition module exhibits poor reusability and is highly susceptible to environmental interference, necessitating its design as a disposable product, which inadvertently increases usage costs. (4) For in vivo detection, aptamers suffer from poor stability, are highly susceptible to degradation by nucleases, and can be rapidly cleared by the kidneys within minutes. Furthermore, when used for in vivo imaging, their metabolic distribution is often concentrated in the liver and kidneys, generating strong background signals that complicate the detection of major organs.^237,267^ In response to these issues of stability and metabolic distribution, not only can chemical modification methods be employed to improve in vivo stability and alter biodistribution, but aptamers can also be directly applied for detection in organs such as the eyes, bladder, and nasal cavity to circumvent high-background abdominal regions.

### Challenges and future perspectives in molecular therapeutics

Although numerous disease-specific aptamers have indeed been developed from the perspective of molecular diagnostics, there are substantial differences between aptamer-based molecular detection and molecular therapeutics, with distinct research focuses and considerations.^23,26^ This discrepancy means that, in most cases, aptamers used for molecular diagnostics cannot be directly applied to molecular therapy. Consequently, it is necessary to rescreen for different diseases under simulated physiological conditions to obtain new aptamers suitable for molecular therapeutic applications. In molecular therapeutics, the stability of aptamers poses a significant obstacle to their development as drugs. This includes issues such as susceptibility to nuclease degradation, extremely rapid metabolism in vivo, strong accumulation in the liver and kidneys, and compromised postbinding stability due to hemodynamic flow.^267,295^ These adverse effects necessitate the use of modification strategies to minimize their impact, including base substitutions with artificial analogs and various chemical modifications of nucleotides.^13^ DNA structure or nanomaterial conjugation can greatly improve aptamer stability, enhance targeting, and modulate metabolism.^13,26^ However, the quality control of nanomaterials and their long-term biosafety require careful consideration during clinical translation. Current research on nucleic acid drugs has primarily focused on RNA, resulting in limited experience with regulatory-compliant production and safety evaluation for DNA-based therapeutics. This knowledge gap contributes to the high costs of preclinical research, underscoring an urgent need for communication with the industrial sector to facilitate the translational pathway for DNA aptamer-based drugs.

Artificial intelligence, machine learning, and computational modeling are significantly accelerating the discovery and optimization of aptamers. During the selection phase, deep learning-based sequence generation and structure prediction models can efficiently identify high-affinity candidates from vast sequence spaces, reducing the time and cost of traditional SELEX experiments. In the optimization stage, computational simulations can elucidate aptamer–target interaction interfaces, guiding key nucleotide mutations and affinity improvement. Meanwhile, multiomics integration and predictive models help evaluate aptamer stability, immunogenicity, and pharmacokinetic properties in vivo. At the clinical development level, AI-driven biomarker matching and patient stratification strategies enhance the precision therapeutic potential of aptamer-based drugs, thereby promoting their systematic translation from basic research to clinical application.

The potential systemic toxicity of ApDCs is one issue that should be considered. The promising in vitro and xenograft results of ApDCs must be considered alongside potential systemic toxicities inherent to targeted chemotherapy. If small-molecule drugs are not securely retained by the target organs, they will pose a toxicity risk to normal organs such as the kidneys, bone marrow, and liver. Furthermore, although ApDCs demonstrate high affinity for cancer cells, off-target binding to low-expression tissues or nonspecific accumulation (e.g., via the reticuloendothelial system or renal clearance) could lead to unintended toxicity. Future studies in immunocompetent, target-expressing models, coupled with detailed biodistribution and dosimetry analyses, will be essential to fully evaluate the therapeutic index and potential mitigation strategies, such as optimized dosing or pretargeting approaches.

## Conclusion

Since their initial report in 1990, research on nucleic acid aptamers has continued for nearly 40 years. Aptamers represent a significant class of molecular tools that are intermediate in size, complexity, and synthetic accessibility between traditional small molecules and proteins. They routinely achieve affinities and specificities comparable to those of antibodies while avoiding the immunogenicity concerns associated with proteins. Additionally, aptamers can be generated more efficiently for a range of targets compared to the high-throughput screening methods used for small molecules.^85,298,312,343^ Although aptamers have shown promise, commercial success with aptamer-based products remains in its early stages, lagging far behind protein drugs. To date, two aptamer-based drugs, Macugen and Izervay, have successfully reached the market. In contrast to antibodies, which benefit from a well-developed commercial infrastructure, the development pathway for aptamers lacks sufficient education, investment, and related knowledge—such as medicinal formulation, pharmacokinetic and pharmacodynamic properties, and toxicity. Additionally, challenges associated with the design and formulation of aptamer-based molecular tools still exist. Overcoming these challenges and fully leveraging the unique attributes of aptamers is vital for their future success in molecular diagnosis and therapeutics.

Despite the limited commercial success of aptamer-based molecular diagnostics and therapeutics, the lessons learned from past failures and successful technological advancements are invaluable for identifying future opportunities in this field. Proof-of-concept studies continue to demonstrate the promising functionality and vast potential of aptamers for diagnosis and therapy. As a unique class of biomolecules, aptamers combine the flexibility of small molecules with the high specificity of antibodies, enabling targeted disease diagnosis and therapy that small drug molecules cannot achieve. These distinctive advantages position aptamers to fill niche markets, including antibacterial and antiviral applications, tissue repair, tumor vaccines, and targeted protein degradation. Furthermore, the development of new screening and chemical modification technologies is rapidly advancing this field. Notably, the emergence of AI technology is quietly transforming the industry’s development model, influencing both research and commercialization.

To achieve the successful clinical translation of aptamer-based technologies, future research should focus on the following directions: (1) Optimized aptamer screening technology. The discovery of high-performance aptamers remains a limiting factor for molecular diagnostics, therapeutics, and industrial applications. Development of clinical application-oriented nucleic acid aptamer screening technology will be a crucial research direction. (2) Precise aptamer structural analysis. Thousands of aptamers have been discovered; however, their true 3D structures have not been well deciphered. Methods for obtaining nucleic acid aptamer crystals and determining their true three-dimensional structures are highly needed. (3) Advanced aptamer stabilization technologies. Ideal aptamer agents should possess precise and stable structures to ensure their structural and functional integrity in the bloodstream. Developing new chemistries and technologies to stabilize aptamer conformation and exploring the conformation–performance relationship would be highly desirable. (4) Versatile aptamer preparation. To meet the demands of precision therapy, multitarget, multifunctional aptamer molecular probes and molecular drugs will become the next research hotspot. (5) Acceptable cost. ApDCs usually have a much lower cost than ADCs. However, high chemical synthesis costs, complex coupling processes, and stringent quality control requirements are the main bottlenecks preventing the large-scale production of aptamer therapeutics. Decreasing the cost of ApDCs could increase the clinical accessibility of aptamer molecular diagnostics and treatment. This may become a new direction for highly interdisciplinary studies to promote the clinical translation and application of aptamers.

The advancement of aptamer technology has led to a surge in molecular diagnostics and therapeutic aptamers. As more researchers and commercial investments focus on rational aptamer development, next-generation aptamer-based diagnostics and therapeutics with enhanced biological functions and pharmacokinetic profiles are highly anticipated. Aptamers have the potential to revolutionize the diagnosis and treatment of diseases, and the market is expected to continue expanding. Many obstacles remain on the path to clinical translation of aptamer drugs, including in vivo metabolic stability, off-target effects, and clinical translation hurdles. The widespread clinical application of aptamer-based therapies requires a coordinated roadmap across multiple dimensions. Scientifically, it is necessary to optimize selection methods, improve in vivo stability, and clarify mechanisms of action. Technically, high-throughput SELEX platforms and AI-driven design tools should be developed. From a regulatory perspective, unified standards for quality and safety evaluation are needed. Commercially, scalable manufacturing and cost control must be advanced, alongside strengthened intellectual property management and translation mechanisms. Coordinating these stages will accelerate clinical translation and industrialization. As research progresses, we can reasonably anticipate a future where aptamers play a pivotal role in precision medicine, offering new hope to patients with various diseases.
